# Estimating between-country migration in pneumococcal populations

**DOI:** 10.1093/g3journal/jkae058

**Published:** 2024-03-20

**Authors:** Sophie Belman, Henri Pesonen, Nicholas J Croucher, Stephen D Bentley, Jukka Corander

**Affiliations:** Parasites and Microbes, Wellcome Sanger Institute, Hinxton, Cambridgeshire, CB10 1SA, UK; Oslo Centre for Biostatistics and Epidemiology, Oslo University Hospital, Oslo, 0372, Norway; MRC Centre for Global Infectious Disease Analysis, Department of Infectious Disease Epidemiology, School of Public Health, White City Campus, Imperial College London, London W12 0BZ, UK; Parasites and Microbes, Wellcome Sanger Institute, Hinxton, Cambridgeshire, CB10 1SA, UK; Department of Biostatistics, University of Oslo, Oslo, 0372, Norway; Helsinki Institute for Information Technology HIIT, Department of Mathematics and Statistics, University of Helsinki, Espoo, Helsinki, 02150, Finland

**Keywords:** approximate bayesian computation (ABC), bacterial populations, genomics, pneumococcus, migration

## Abstract

*Streptococcus pneumoniae* (the pneumococcus) is a globally distributed, human obligate opportunistic bacterial pathogen which, although often carried commensally, is also a significant cause of invasive disease. Apart from multi-drug resistant and virulent clones, the rate and direction of pneumococcal dissemination between different countries remains largely unknown. The ability for the pneumococcus to take a foothold in a country depends on existing population configuration, the extent of vaccine implementation, as well as human mobility since it is a human obligate bacterium. To shed light on its international movement, we used extensive genome data from the Global Pneumococcal Sequencing project and estimated migration parameters between multiple countries in Africa. Data on allele frequencies of polymorphisms at housekeeping-like loci for multiple different lineages circulating in the populations of South Africa, Malawi, Kenya, and The Gambia were used to calculate the fixation index ($F_{st}$) between countries. We then further used these summaries to fit migration coalescent models with the likelihood-free inference algorithms available in the ELFI software package. Synthetic datawere additionally used to validate the inference approach. Our results demonstrate country-pair specific migration patterns and heterogeneity in the extent of migration between different lineages. Our approach demonstrates that coalescent models can be effectively used for inferring migration rates for bacterial species and lineages provided sufficiently granular population genomics surveillance data. Further, it can demonstrate the connectivity of respiratory disease agents between countries to inform intervention policy in the longer term.

## Introduction

Inferring migration events in natural bacterial populations between geographically separated regions is generally challenging since bacteria cannot be tagged in the same way as, for example, animals. For bacteria which colonize humans the rate of migration will be a complicated function of the host mobility and ecological factors influencing the success of onward transmission, such as the level of hygiene, use of antibiotics, and vaccination campaigns in the host populations. Progress identifying large-scale migration patterns among bacteria has been primarily made for species or strains more likely causing acute infections and serious illnesses, such as cholera (Okoro *et al.* 2012; Comas *et al.* 2013; Domman *et al.* 2017; Lassalle *et al.* 2023). However, genetic epidemiology has also been used to elucidate the spread of multidrug resistant (MDR) strains of *Streptococcus pneumoniae* (the pneumococcus) across different global regions. For such commonly asymptomatic bacteria, these studies are highly reliant on comprehensive sampling (Croucher *et al.* 2014; van Tonder *et al.* 2015; Moreno and Araque 2018). Overarchingly, variable sampling strategies between countries, poor approximation of between-country mobility, and the large time scales of between-country pathogen spread can hinder definitive estimates of the weight and direction of between-country spread.

Global genomic sampling as part of the Global Pneumococcal Sequencing (GPS) Project demonstrates that some lineages and serotypes of this bacterium circulate locally, while others are spread globally. With the exception of the aforementioned MDR strains, the time scales of this spread and the frequency, or direction of migration between specific countries leading to the extant lineage distributions remains unclear (Gladstone *et al.* 2019). While we can use time resolved phylogenies to infer that genome pairs from countries in South Africa and countries elsewhere in Africa become increasingly similar with increasing divergence times, this is still, overall, much less likely than pair similarity within country (Belman *et al.* 2023). Informing coalescent models with true case count data can reduce the impact of geographic sampling bias, but for an endemic, often asymptomatic pathogen this remains difficult (Layan *et al.* 2023). Our previous work used human mobility data from Meta (Maas 2019) to build a model inferring bacterial movement (Belman *et al.* 2023), but could provide limited information about the direction of spread. Further, reliable between-country human mobility data is scarce for continents such as Africa, so similar approaches have limited use in this context (Gabrielli *et al.* 2019; Deutschmann *et al.* 2022).

In this work, we quantify between-country pneumococcal migration among four African countries. Due to the lack of direct observations of between-country migration, we are unable to use typical Bayesian techniques. However, population genomics-based surveillance data from multiple countries provides an opportunity to consider quantification of migration rates via coalescent models and likelihood-free inference (LFI) (Wegmann *et al.* 2009; Aeschbacher *et al.* 2013). In particular, we use Approximate Bayesian Computation (ABC), which is a type of LFI in which we compare population summary statistics between simulated and real genomic data to determine (in this case) patterns of pneumococcal migration.

We can explore the impact of different evolutionary parameters on population samples using software packages which simulate coalescing populations (Ewing and Hermisson 2010; Kern and Schrider 2016; Kelleher and Lohse 2020). msprime is an adaptation to the classical neutral ms simulator which includes demographic parameters such as population size and migration (Hudson 2002). Such a simulation-based inference framework (Sisson *et al.* 2018) enables inference of the unknown migration parameters without access to a closed-form expression for the data under any particular set of parameters assumed to govern the migration process. Given the generality of the problem of migration quantification for microbes, our approach is of wider interest beyond the pneumococcal case considered in detail here.

## Materials and methods

### Population divergence summary statistics

Rather than comparing the entire nucleotide diversity of genes and genomes within and between populations one can summarize the diversity using relevant statistics derived from population genetics theory under neutrality. The fixation index ($F_{st}$) was first developed by Wright (1949) in 1949 to describe randomly drawn alleles in one population as compared to the total sampled population. There have been many adaptations to this but the Weir & Cockerham (WC) $F_{st}$ (Weir and Cockerham 1984) is the most commonly used today and describes the total population as the most recent ancestral population. The WC $F_{st}$ is a parameter describing the evolutionary process of drift rather than a statistic of observed samples and assumes equal drift across populations. When the $F_{st}$ for each population is different the WC estimator becomes a function of the ratio of sample sizes between them rather than true divergence. Further WC $F_{st}$ suffers from the “star phylogeny” assumption in that all populations independently descended from the same ancestor. Weir & Hill adapted the WC $F_{st}$ to estimate population-specific values and Hudson et al. adapted this to estimate the $F_{st}$ between populations in 1992 (Selander and Hudson 1976; Hudson *et al.* 1992; Holsinger and Weir 2009). Hudsons $F_{st}$ is more robust to variable sample sizes and variable $F_{st}$ values across populations (Bhatia *et al.* 2013). Simply Hudsons $F_{st}$ can be described by Weir & Hills single population $F_{st}$ estimate.


(1) $$\begin{array}{cc} & E[p_{i}^{s}\;|\;p_{anc}^{s}]=p_{anc}^{s}\\ & \;\;\;Var(p_{i}^{s}\;|\;p_{anc}^{s})=F_{ST}^{i}\cdot p_{anc}^{s}(1-p_{anc}^{s}) \end{array}$$

where $p_{i}^{s}$ is the allele frequency in population *i* at SNP *s*, and $p_{anc}^{s}$ is the frequency of the same allele at that SNP *s* in the ancestral population and $F_{st}^{i}$ is the population-specific $F_{st}$ for population *i*. Hudsons estimate combines these to estimate the $F_{st}$ for a pair of populations to be


(2) $$F_{st}=\frac{F_{st}^{1}+F_{st}^{2}}{2}$$

where $F_{st}^{1}$ is population 1 and $F_{st}^{2}$ is population 2.

### Inference strategy overview

#### Simulator

We used msprime (Kelleher and Lohse 2020; Nelson *et al.* 2020; Baumdicker *et al.* 2022) as the simulator in this framework. msprime is a software package which simulates the coalescent process for thousands of genomes. It can incorporate recombination, mutation, and migration between demes. It outputs phylogenetic trees representing the population, onto which mutations can be imputed and summary statistics calculated. These summary statistics are then compared to summary statistics from the real data. The coalescent simulation requires specific parameters. For the two-deme model, we included geographic demes *A* and *B* and initial population size for each deme ($P_{A}$, $P_{B}$). The sample sizes drawn from each population ($samp_{A}$, $samp_{B}$) are included as parameters and crucially, we include the inferrable parameters for the migration rates asymmetrically from deme *A* to deme *B*($mig_{A,B}$), and the inverse ($mig_{B,A}$). We also include a mutation rate (*θ*). We specified these parameters scaling the mutation rate to the length of genome we input and down-sampled the true population sizes from each country for computational efficiency. We included the infinite sites model in this framework which rather than allowing only a finite number of mutations persite allows an infinite number across continuous space whereby no site mutates twice (Kimura 1969; Ma *et al.* 2008) (Table 1). We employ replicates, both for the simulator and the real-data, to reduce variability around the summary statistic estimate (Table 1).

**Table 1.** Parameter summary for inference validation.

Parameters	Truth		Simulated input	
Deme Population Sizes	South Africa	60.04 M	South Africa	6,000
	Malawi	19.65 M	Malawi	2,000
	Kenya	54.99 M	Kenya	5,000
	The Gambia	2.487 M	The Gambia	1,000
Mutation Rate (*θ*)	1.57e−06		2.50e−05	
Discrete Genome	True (finite sites model)		False (infinite sites model)	
Sequence Length	2 Mbp		500	
Migration Rate	?		$mig_{ab}=0.6$	
			$mig_{ba}=0.1$	

Estimates of true parameter values for the deme population size, mutation rate in sites/per-year, whether there is a discrete genome, and sequence length for left) truth and right) within inference validation msprime simulation framework.

#### Parameter inference algorithm

LFI methods such as ABC were originally developed to do statistical inference with a very large number of synthetic observations from the simulator, rejecting nonconforming observations and keeping only observations close the observed data (Sisson *et al.* 2018). However, with a complex process involving multiple unknown parameters millions of simulations are often necessary to infer the required parameter using the basic rejection algorithm. A state-of-the-art ABC method such as the Bayesian Optimization for Likelihood-Inference (BOLFI) algorithm employs active learning to reduce the required computer simulation multiple fold by focusing only on the relevant parts of the parameter space. This can be done using a probabilistic model such as a Gaussian Process to model the relationship of parameters and the discrepancy between the observed data and synthetic data, and seeking to minimize this Gutmann and Cor (2016). BOLFI provides us with a surrogate likelihood function that we use in a Bayesian framework along with the parameter prior distribution to obtain the posterior distribution. In practice, a sample is drawn from the posterior using, e.g. MCMC sampling to calculate posterior means and other summarizing estimates.

#### Model validation: recapturing simulated data

##### Two-Deme Model

**Fig. 1.**
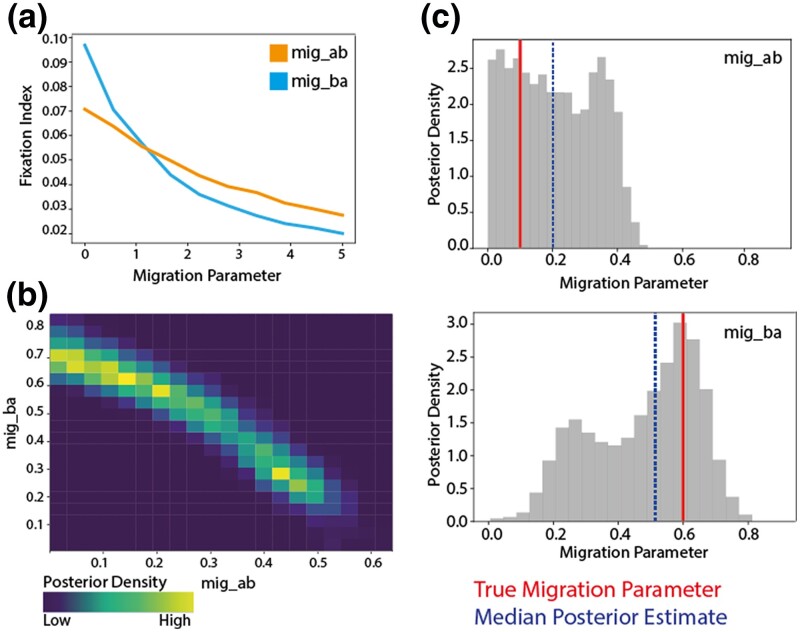
Recapturing input migration parameters with a two-deme model. a) Prior predictive analysis testing the sensitivity of our summary statistic, the fixation index, to our parameter estimates for each parameter in an asymmetric model. Initial populations sizes for A and B of 6,000 and 2,000 with 600 sampled from each. Other parameters include a sequence length of 500, *θ* set to 2e−5, and 500 replicates. The migration parameter is divided by 5,000 for scaling. b) The overlapping posterior density migration parameter estimates for migration parameter “$mig_{ab}$”—from a population A (initial population size 6,000) to population B (initial population size 2,000) and “$mig_{ba}$” from population B to population A. The “true” input parameters were $mig_{ab}$ = 0.1 and $mig_{ba}$ = 0.6. c) The posterior densities visualized individually for each parameter (gray), the true input parameter is indicated by the red vertical line while the median posterior estimate is indicated by the blue dashed line.

To validate this inference framework, we first conducted prior predictive analysis to determine the sensitivity of the summary statistic, $F_{st}$, to a range of migration parameters. We maintained initial populations sizes for A and B of 6,000 and 2,000 with 600 sampled from each. Other parameters include a sequence length of 500, *θ* set to $2e^{-5}$, and 500 replicates. We divided the migration parameter by 5,000 for scaling (Fig. 1a). We also evaluated the sensitivity of the $F_{st}$ to other parameters in the simulator framework including the initial and sampled population sizes, and the mutation rate (Supplementary Fig. S1a–c). We input specific asymmetric migration parameters ($mig_{A,B}$ and $mig_{B,A}$) between two demes (*A* and *B*) and attempted to recapture them using simulated data. For our validation, we again set the mutation rate to $2.5\cdot 10^{-5}$ , set the number of replicates to 500, sequence length of 500, $samp_{A}=600,samp_{B}=600,$  $P_{A}=6,000,P_{B}=2,000$, corresponding to the population sizes of each country as estimated from LandScan (Rose *et al.* 2020) (Table 1). We use the infinite sites model by setting discrete genome to FALSE thus including infinite possible SNP sites. We calculate the $F_{st}$ for each simulation and use nine quantiles at 0.1 increments across replicates for the summary statistic. We use uniform priors $mig_{A,B}∼\text{Unif}[L,U]$ and $mig_{B,A}∼\text{Unif}[L,U]$ to calculate the posterior distributions where *L* represents the lower bounds and *U* represents the upper bounds. We input 0.6 and 0.1 for $mig_{A,B}$ and $mig_{B,A}$, respectively (Fig. 1b–c; red line, Supplementary Fig. S2). The estimated parameters for $mig_{A,B}$ and $mig_{B,A}$, respectively, were 0.207 [95% Confidence Intervals (CIs) 0.011–0.422] and 0.477 (95% CIs 0.164–0.721) (Fig. 1b–c ; blue-dashed line, Supplementary Fig. S2).

We repeated this validation varying the initial population size of each deme relative the true population size of each country (Table 1, Supplementary Fig. S2). There is colinearity between the asymmetric parameters. The model is able to resolve this and recapture the correct peak for each parameter.

##### Four Deme Model

We validate a symmetric four-deme model in which the weight, not the direction of migration, is estimated between all four-deme pairs within one model. We conducted prior predictive analysis to determine appropriate bounds for the uniform prior (Supplementary Fig. S3) and the impact of each parameter on all other parameters. We found that the parameters were not independent of each other and altering one migration parameter impacted the $F_{st}$ of another (Supplementary Fig. S3) implying that migration between two countries may impact the migration estimates in a third or fourth additional country. This relationship is expected due to the migration across all demes included in the simulation which is inherent to our framework.

To validate the four-deme model, we input six migration parameters ($mig_{a}b=2.5,mig_{a}c=2.5,mig_{a}d=2.5,mig_{b}c=1.5,mig_{b}$  $d=1.5,mig_{c}d=0.5$) and were able to recapture them within the symmetric model. The true migration parameter is indicated by the red vertical line while the estimated median parameter is indicated by the blue-dashed vertical line. The input population sizes for each of the demes scale to the true population size and are indicated in Supplementary Fig. S4. All other parameters are consistent with those described in Section “Two Deme Model” however only a single migration parameter is input for each deme pair.

### Application to a pneumococcal dataset

#### Isolate culture and sequencing

**Fig. 2.**
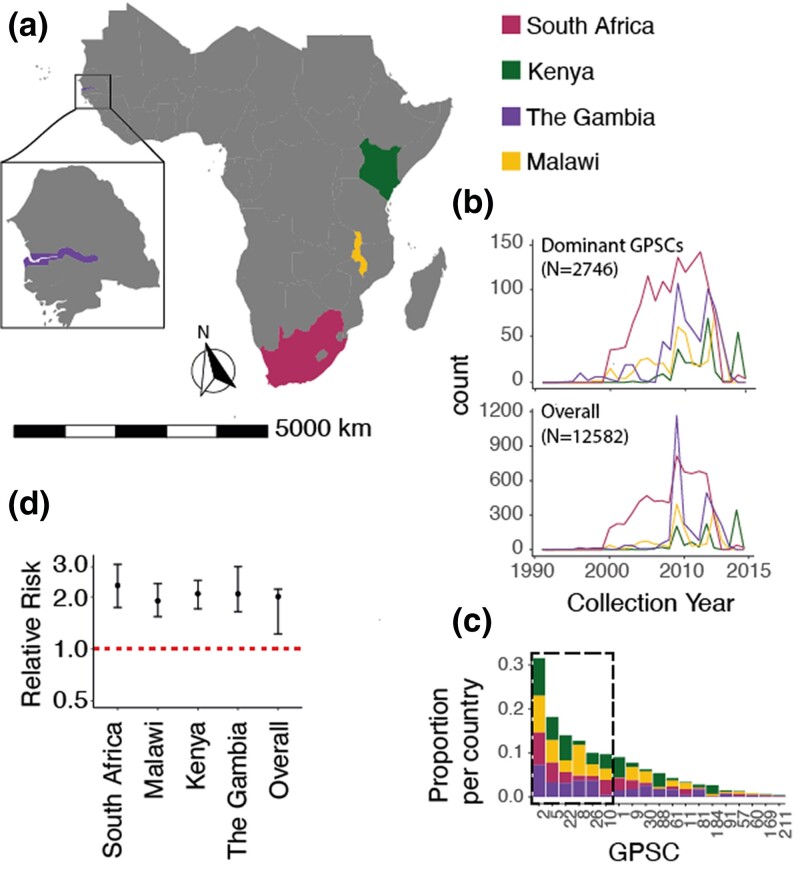
Population composition of four demes. a) Map of isolation location of pneumococcal genomes including South Africa (pink), Kenya (green), The Gambia (purple), and Malawi (yellow). b) Bottom) The isolates were collected from 1990 to 2014 and the total dataset included N=12,582 isolates Top) subsetting by the “Dominant GPSCs” those spanning all four demes and comprising >1% of the GPSCs in each country included six total GPSCs highlighted in c) which shows the proportion of total GPSCs each GPSC comprised in each country. The dominant six are outlined in a gray box. d) The relative risk of similarity within country as compared to between countries at the lineage level for each country and the overall risk of being the same GPSC from the same country compared to different countries.

We included pneumococcal genomes from four Sub-Saharan African countries including South Africa (N=6,919), The Gambia (N=3,090), Malawi (N=1,612), and Kenya (N=961) from the GPS Project in our initial dataset (GPS 2022) (Fig. 2a, Table 5). The isolates were collected between 1990 and 2014 (Fig. 2b), comprised 360 GPSCs and 83 different serotypes, and were randomly selected for sequencing (Fig. 2c). *“Country”* will be used interchangeably with *“Deme”* throughout this manuscript. We will also interchangeably use *“GPSC”* and *“lineage”*.

We calculated the relative risk of GPSC similarity by country as per the method described in Belman *et al.* (2023). To determine whether there are distinct GPSCs circulating in each country we calculated the risk that a pair of isolates sampled from the same country would be the same GPSC as compared to a pair of isolates selected from each country and another country (Fig. 2d). There was a 2.02 (95%CIs 1.23–2.23) times increased risk of a pair being the same GPSC when from the same country as compared to pairs from different countries indicating that there were distinct GPSCs circulating in each.

These pneumococcal isolates were selectively cultured on BD Trypticase Soy Agar II with 5% sheep blood (Beckton Dickinson, Heidelberg, Germany) and incubated overnight at $37^{\circ }C$ in 5% $CO_{2}$. Genomic DNA was then extracted manually using a modified QIAamp DNA Mini Kit (QIAGEN, Inc., Valencia, CA) protocol. As part of GPS, pneumococcal isolates were whole-genome sequenced on the Illumina HiSeq platform to produce paired-end reads with an average of 100–125 bases in length and data were deposited in the European Nucleotide Database. Whole-genome sequence data were processed as previously described (Gladstone *et al.* 2019).

To control for population structure, and be sure the parameters we were estimating were not just due to lineage diversity in each country, we included only GPSCs which are at greater than 2% prevalence overall in the population (11 GPSCs), we then limited to only those with isolates present in each of the four countries (7 GPSCs), and finally we restricted based on those GPSCs which comprised >1% of the remaining number of isolates in each country. Ultimately we included six “Dominant GPSCs” (GPSC2, GPSC5, GPSC8, GPSC10, GPSC22, and GPSC26) which included a total of 2,746 genomes.

#### Neutral gene selection

We selected genes which are less impacted by evolutionary selection processes due to their less than 0.1 median IgG binding affinity, and “nonantibody binding” status in Croucher *et al.* (2017). The selected genes are present with at least a 99% frequency across our dataset. Across the whole dataset we included two groups of “neutral” genes: (1) a subset of 341 genes which were nonABT, and (2) 84 genes which had <0.1 median IgG binding affinity *and* were nonABT. For the by-GPSC analysis, we selected 81 and 355 “neutral” genes which were core across all six Dominant GPSCs and fit the same criteria as above.

#### Pairwise distances between genes

We built genome alignments for the previously described sets of genes utilizing a combination of Panaroo (Tonkin-Hill *et al.* 2020) and BioPython (Chapman and Chang 2000). We calculated pairwise distances from every genomes to every other genomes using both Hamming (Supplementary Fig. S5a) (Hamming 1950) and Jaccard distances (Supplementary Fig. S5b) (Jaccard 1912; Murphy 1996) grouping the genomes by isolation country. If there were distinct, qualitative differences between countries one would expect clear divergent blocks of similarity (lighter color) along the diagonal of the pairwise distance plots. We used scikit-allel (Miles *et al.* 2021) for all pairwise distance calculations.

At the population level (N=12,582) the homogenous color across both Jaccard, and Hamming distance plots is representative of mixed populations across the countries. Despite the two times higher probability of a pair being the same GPSC when from the same country as compared to pairs from different countries there are still many similar GPSCs between them (Supplementary Fig. S5).

#### Controlling population structure

##### GPSC Level

To control for population structure, we interrogated each Dominant GPSC (N=2,746), for the pairwise distances between genes across countries. We only included genes which were present in all four countries and across the Dominant GPSCs (N=81: nonABT & <0.1 IgG Binding; N=355: nonABT). The 81 selected genes had a median gene length of 555 bp (95% CIs 207–1809) (Supplementary Fig. S6).

We grouped the alignments by country to see if there were qualitatively distinguishable differences within versus between country. Again we calculated Hamming distances for 81 and 355 (Supplementary Fig. S7) genes. We repeated this using Jaccard distances for both gene sets (Supplementary Fig. S8). Jaccard and Hamming distances resulted in similar patterns.

##### Linked SNP Sites

Coselected sites may exacerbate the signal within countries due to recombination, and thus mask the migration signal between countries. To exclude coselected sites, we calculated the mutual information score (MI) across the concatenated neutral genes both overall and for each GPSC. We visualized these using SpydrPick (Pensar *et al.* 2019) to understand the relationship across all 81 genes biallelic SNP sites (Table 2, Supplementary Fig. S9). The majority of sites with high, direct MI scores were within 1 kb of each other. GPSC2 and GPSC8 have high linkage across many nucleotide distances which span selected genes. These are invasive lineages and undergo less recombination than other lineages. This is demonstrated by each only comprising a single PCV13-type serotype, serotypes 1 and 5, respectively. You can explore each lineage phylogeny at the Microreact web server (Table 4).

**Table 2.** Biallelic SNP count for each GPSC.

GPSC	Total SNPs	Excluding 0.5 Threshold	Excluding 0.05 Threshold
2	968	214	171
5	1,745	336	161
8	580	78	62
10	1,221	176	104
22	1,469	294	132
26	543	92	60

Including total number of biallelic SNPs, total excluding all within a 1 kb window upstream with an $r^{2}>0.5$, and total excluding all within a 1 kb window upstream with an $r^{2}>0.05$.

Given the distribution of gene length when the distance between SNP sites exceeds approximately 1.8 kb the sites are separated by more than one gene length (Supplementary Fig. S6) resulting in ultimately fewer genes being included.

We controlled for coselection between SNPs with a strict correlation threshold minimum of 0.05 $r^{2}$ and a more flexible minimum correlation of 0.5 $r^{2}$. We used bcftools +prune to remove all SNPs with an $r^{2}$ greater than the threshold within a 1 kb upstream window and repeated our two deme migration analysis for each GPSC between South Africa and Malawi (Table 2). A 1kb window encompasses the entire length of the majority of genes included (Supplementary Fig. S6). We proceeded with the 0.5 relatedness threshold as it excluded fewer SNP sites but maintained similar estimates as the stricter threshold (Supplementary Fig. S10).

#### Fixation index

To quantify the divergence between each location, we then calculated $F_{st}$ overall and by GPSC using tskit (Kelleher and Lohse 2020; Baumdicker *et al.* 2022; Nelson *et al.* 2020). We compared the SNP $F_{st}$ between Weir-Cockerham and Hudsons $F_{st}$ using tskit within the coalescent simulation software msprime (Kelleher *et al.* 2016; Nelson *et al.* 2020; Baumdicker *et al.* 2022). Hudson’s $F_{st}$ is calculated in tskit:


(3) $$F_{st}=\frac{1-2\cdot (d(X)+d(Y))}{\left(2\cdot d(X,Y)+d(X)+d(Y)\right)}$$

where *X* and *Y* and d(X) and d(Y) are the populations and diversity of those populations, respectively, and d(X,Y) is the shared diversity of both populations. A higher $F_{st}$ corresponds to a more divergent, separate population, while a lower $F_{st}$ corresponds to a more highly mixing population, also known as panmictic.

We compare the $F_{st}$ estimates from Weir & Cockerham and Hudson and find them to be largely linear with some overestimates by WC. Due to the uneven sample sizes of our populations we proceed with Hudson’s estimate (Supplementary Fig. S11).

We calculate the $F_{st}$ across all genomes between each of the four demes for each GPSC. We repeat this including 81 neutral genes, 355 neutral genes, and the *pbp* genes as a control for selection (Supplementary Fig. S12). We find variable estimates both across GPSCs and between countries. Notably GPSC2 and GPSC8 have higher estimates overall than the rest of the GPSCs in both the 81 and 355 gene comparisons (Supplementary Fig. S12a–b). We included the *pbp* genes as these genes confer resistance to *β*-lactams and are under significant selective pressure. Given variable selective pressures across countries they would be expected to have different cross-country diversity patterns than our “neutral” selected genes. We do see differences between the *pbp* gene divergence as compared to the divergence patterns we see when including the “neutral” genes. This is reassuring as we expect a different pattern as a result of AMR selection. The $F_{st}$ for the 355 genes was less informative with regards to the different populations. Hereafter we only include the 81 gene analysis by GPSC. All analyses and visualizations was conducted in Python v3.9.13 and R v3.6.1.

### Overall between-country migration risk

While the above framework identifies migration parameters when migration occurs, it does not account for the actual probability of movement between countries as compared to movement within a country. To address this, we applied a simple relative risk framework incorporating divergence time between genome pairs. We included those most prevalent GPSCs across the dataset GPSC2 (N=904), GPSC5 (N=473), GPSC10 (N=306). We created reference genomes for each GPSC using ABACAS to order the contiguous sequences (contigs) from a representative of each GPSC mapped to *Streptococcus pneumoniae* (strain ATCC 700669/Spain 23F-1) [EMBL accession: FM211187]. Any contigs which did not align were concatenated to the end. We multiply mapped all genomes from each GPSC against these references, respectively, using a custom mapping, variant calling, and local realignment around indels pipeline using bwa-MEM (Li and Durbin 2009) and samtools mpileup (Li *et al.* 2009). We built trees masking recombination regions using Gubbins (Croucher *et al.* 2015) with RAxML (Stamatakis 2014) and a general time reversible (GTR) evolutionary model. We converted branch length to time using BactDating with a mixed gamma, relaxed clock model (Didelot *et al.* 2018).

We compare the location (*loc*) and label (*G*) (genetic similarity) of pairs of sequences (*i*,*j*) that were collected around the same time (*t*). This approach has been shown to be robust to substantial biases in timing and location of isolate collection. To determine at what divergence time it became equally likely that a pair of genomes were within the same country as between different countries, for each country, we constructed pairwise matrices comparing every isolate to every other isolate (*N* Pairs = 1,683). We then determined the proportion of genomes at each divergence time across rolling 10-year time windows within a country as between countries. Dividing the proportion which are within the same country by the proportion between countries gives the relative risk.

In this case, the numerator contains the ratio of pairs which are at each divergence time, collected within 10 years of each other *t*, from the same country *loc*, over the total number of pairs collected within 10 years of each other t≤10years from the same country. The denominator is the ratio of pairs which are within each divergence time *G*, collected within 10 years of each other, from different countries ($L_{ref}$) over the total number of pairs collected within 10 years of each other from different countries. Geographic distances were calculated based on the centroid coordinates of each province (Equation 4).


(4) $${RR}_{loc}(g1,g2)=\sum_{i=1}^{n}\sum_{\;j\neq i}^{n}\frac{\frac{\left({loc}_{i}={loc}_{\;j}\cap t_{ij}\leq 1\;year\cap G_{i}=G_{\;j}\right)}{\left({loc}_{i}={loc}_{\;j}\cap t_{ij}\leq 1\;year\right)}}{\frac{I({Lref}_{n}={Lref}_{n}\cap t_{n}\leq 1\;year\cap G_{i}=G_{\;j})}{I\left({Lref}_{n}={Lref}_{n}\cap t_{n}\leq 1\;year\right)}}$$

To quantify uncertainty, we used a bootstrapping approach where in each bootstrap iteration we randomly sampled with replacement the isolates before recalculating the statistic. We report the 2.5 and 97.5 percentiles from the resulting distribution.

## Results

### Inference strategy

In brief, to estimate between-country migration parameters we developed a framework which uses summary statistics to characterize a sampled pathogen population and compares them to corresponding statistics from a simulated pathogen population under a given coalescent model. We use the Hudsons fixation index ($F_{st}$) as the summary statistic in this model. We employ several key pieces of software including msprime to simulate a coalescing population, and the Engine for Likelihood-free Inference (ELFI) (Lintusaari *et al.* 2018) to compare the simulated populations with the observed population data. Broadly, our strategy falls under the Approximate Bayesian Computation (ABC) paradigm (Sisson *et al.* 2018).

Using this framework, we develop two models for quantifying migration. The two-deme model compares pairs of countries and determines the asymmetric migration parameters between them (the amount of migration from country A to country B and the reverse). The other model is a four-deme model quantifying symmetric migration parameters between four countries, encapsulated in six rate parameters.

### Application to a pneumococcal dataset

We implement both of these models using genomes from the GPS project (Fig. 2a, Table 5) (GPS 2022). We included GPSCs (also referred to as lineages throughout this paper) representing pneumococcal between-country variation in neutral genes and approximately unlinked SNP sites. We included 12,582 genomes for initial exploration but ultimately reduced this to six “Dominant GPSCs”(GPSC2, GPSC5, GPSC8, GPSC10, GPSC22, and GPSC26) (N=2,746 genomes) from South Africa, The Gambia, Malawi, and Kenya for the migration models.

The isolates were collected between 1990 and 2014 (Fig. 2b), comprised 360 GPSCs and 83 different serotypes, and were randomly selected for sequencing (Fig. 2c). *“Country”* will be used interchangeably with *“Deme”*throughout this manuscript. We will also interchangeably use *“GPSC”* and *“lineage”*(Fig. 2b–c, Tables 3 and 4).

**Table 3.** The number of genomes for each country and GPSC.

	GPSC10	GPSC2	GPSC22	GPSC26	GPSC5	GPSC8	Total
South Africa	232	506	173	73	309	82	1,375
Malawi	41	136	43	44	82	112	458
The Gambia	13	224	96	112	102	112	659
Kenya	32	82	55	25	51	9	254

Including only GPSCs which were >2% prevalence overall, present in all four countries, and present at >1% prevalence in each country.

**Table 4.** Interactive phylogenetic trees for each GPSC presented in the web server Microreact.

GPSC	Interactive phylogeny and metadata hosted on Microreact
2	https://microreact.org/project/7pCYuuCbZ4uU4PeAqywV4H-gpsc2g3
5	https://microreact.org/project/tGmtFEajonz69a1bkrzDve-gpsc5g3
8	https://microreact.org/project/iP7FLWdt6yF5wTGzV9bi7o-gpsc8g3
10	https://microreact.org/project/gmTCuGfsq97gXQFtULurx9-gpsc10g3
22	https://microreact.org/project/4MbjBD1eXrJqF4iqjJ6k2N-gpsc22g3
26	https://microreact.org/project/arus4778ohoTjwKf4Uhhm7-gpsc26g3

**Table 5.** Country summary for between-deme migration analysis.

Country	*N*	Vaccine	Dosing	%NVT	%Children
		Introduction	Schedule		<5
South Africa	6,919	PCV7: 2009	2+1	31.91	65.2
		PCV13: 2011			
The Gambia	3,090	PCV7: 2009	3+0	56.70	46.4
		PCV13: 2011			
Malawi	1,612	PCV13: 2011	3+0	42.68	49.6
Kenya	961	PCV10: 2011	3+0	53.28	80.3

Description of the four countries between which we estimated migration including number from each country, years of vaccine introduction and dosing schedule, the proportion of the dataset for each country which comprised NVTs and the percent of isolates from each country which were from children <5.

We included 81 “neutral” nonantibody binding-type genes selected from the pangenome wide immunological screen conducted by Croucher *et al.* (2017). These genes were core (minimum 99% prevalence) across each of the Dominant GPSCs. Further, we excluded linked SNP sites with a greater than 0.5 $r^{2}$ relatedness threshold. We explored the Jaccard and Hamming distances between genomes for both the 81 and 355 genes and found clear boundaries between the country clusters with Hamming distances ranging from 0 to 0.5. Some GPSCs had clear distinguishable divergence between specific countries while other country pairs were very similar by Hamming distance (Supplementary Fig. S7 and Fig. S8).

### Estimating the weight and direction of migration for country pairs

To determine the symmetry of migration across all four demes included in this model (South Africa, The Gambia, Kenya, and Malawi) we considered six separate models, one for each GPSC, estimating the migration parameters between deme pairs. We used a uniform prior with bounds corresponding to the sensitivity of the simulation demonstrated in the $F_{st}$ sensitivity analysis (Fig. 1a).

We implemented the two-deme model to estimate migration parameters between each pair of demes asymmetrically given the 81 concatenated neutral genes with linked sites removed. We fit the model using BOLFI approximation and drew a posterior sample from it. We ran three independent implementations. We successfully estimated 10 of the 12 asymmetric migration parameters for all deme pairs by GPSC (Supplementary Fig. S13, Fig. S14, Table S1). Parameter estimates for GPSC10 and GPSC26 between South Africa and Kenya did not settle on a single migration parameter in either migration direction due to the bimodal distribution of the posteriors and a colinear relationship between the parameters. A migration weight which was higher from South Africa to Kenya than from Kenya to South Africa resulted in a similar model as the reverse; as such the model was unable to resolve these parameters (Supplementary Fig. S14). Resolving the peak which is “best” is likely futile in that both peaks are equally likely to be true (Supplementary Fig. S14).

**Fig. 3.**
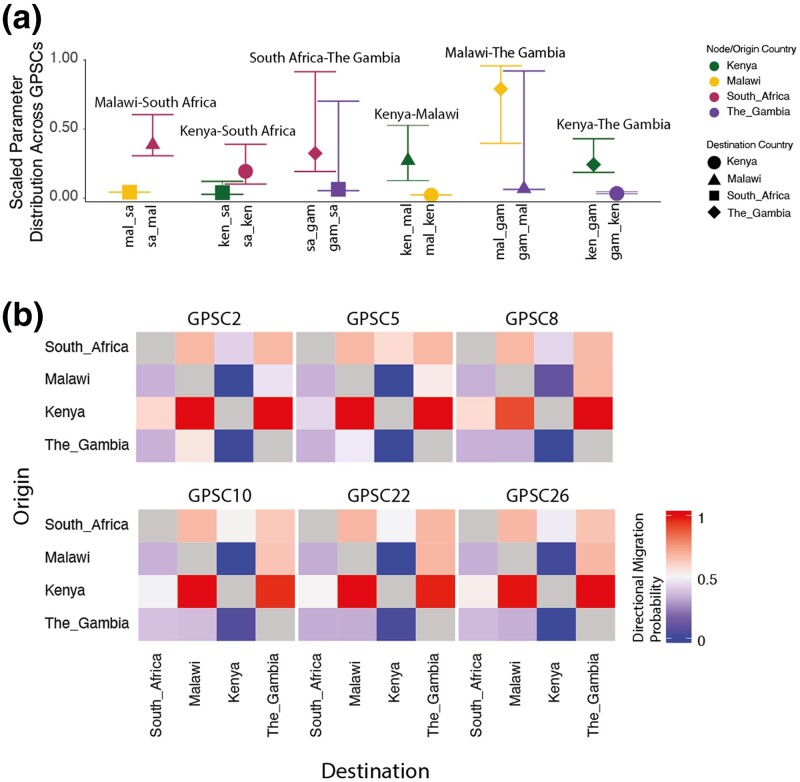
Migration parameters summary estimates a) The scaled distribution of migration probabilities for each deme across GPSCs grouped by deme pair and colored according to country of origin and shaped according to destination. b) Migration parameters directional probability estimates between each deme pair. Colored by the probability of migration between each deme pair. where red is >50% migration probability and blue is <50% migration probability.

To summarize and compare migration between demes across GPSCs, we divided the median posterior value by the maximum bound to find the probability of migration for each parameter. We then found the distribution of migration probabilities for each deme where the confidence intervals represent the distribution of values across GPSCs (Fig. 3a). The difference between the estimates for each deme pair is the relative probability of migration. For example, the pneumococcal populations of South Africa and Malawi are consistently better explained by 50% more migration from South Africa to Malawi than the reverse, while the distribution between Malawi and The Gambia is explained differently across GPSCs with on average more migration from Malawi to The Gambia. The exception to this deme pair is in GPSC2 where the reverse is true (more migration from The Gambia to Malawi) (Fig. 3b, Supplementary Table S1).

#### Weight

To normalize the migration parameter estimates for each GPSC, we found the mean parameter estimate across all posterior samples for each GPSC ($mig_{gpsc}$). We then calculated the relative migration parameter estimate by dividing each parameter by the GPSC-specific parameter ($\frac{mig_{ab}}{mig_{gpsc}}$). We first group these by deme to determine if there are consistent deme-wise patterns(Supplementary Fig. S15a) and then group them by GPSC to identify the differences across demes (Supplementary Fig. S15b, Table S1).

#### Direction

We estimated the probability of directional migration for each GPSC and deme pair by identifying the percentage of posterior migration parameter estimates for $mig_{ab}$ which were greater than those for $mig_{ba}$. We then grouped these into high (≥0.6), medium (0.4–0.6), or low (≤0.4) migration probability. There were two migration patterns. Pattern one is seen in GPSC26, GPSC22, GPSC10, and GPSC8 in which there was *only* symmetric migration between Kenya and South Africa and asymmetric between all other deme pairs. (Fig. 3b). Pattern two applied to GPSC5 and GPSC2 and included symmetric migration between Kenya and South Africa as well as between Malawi and The Gambia, where all other pairs had asymmetric migration patterns (Fig. 3b). Across all GPSCs there was a higher migration probability from Kenya to Malawi and Kenya to The Gambia than from either of those to Kenya; and a higher migration probability from South Africa to Malawi and South Africa to The Gambia than from either of those to South Africa. Considering South Africa and Kenya have the highest population sizes (60.04 Million and 54.99 Million, respectively, in 2019) this implies that the higher the population size, its relative contribution to between pair migration is likely to be higher (Table 1). For those GPSCs with migration pattern one, we estimated a higher migration probability from Malawi to The Gambia, again in line with this hypothesis (Supplementary Table S1). The consistent directional patterns between GPSCs from independent models is reassuring and helps to validate our framework.

#### Demographic contribution to migration

Using the raw parameter estimates from all two-deme asymmetric models, we interrogated whether the origin population size (*α*), the destination population size (*β*), or the distance between deme pairs at the centroid (km) (*γ*), had a larger contribution to the migration parameter estimates (*θ*). We fitted four logistic models, one for each parameter, and one model including all three parameters (Equations 5–8). In the model encompassing all three parameters a greater destination population size was significantly associated with the parameter estimate ($P=1.07e^{-06}$), while the origin population size (P=0.606), and distance (P=0.877) were not associated (AIC = 150.13)(Equation 5).


(5) glm(θ∼α+β+γ)

In both the overall model (Equation 5) and the individual model (Equation 7), a greater destination population size was again significantly associated with a smaller migration parameter (Overall: Coefficient −0.021, $P=1.07e^{-06}$, AIC = 150.13; Individual: Coefficient =−0.021, $P=1.02e^{-08}$, AIC=146.66). The distance between countries was marginally associated (Coefficient 1.014e–04, P=0.0354; AIC = 176.05), but surprisingly in that a larger distance resulted in a higher migration parameter. This is largely driven by GPSC26 where the highest migration parameters are associated with The Gambia which is the most distant country from Kenya, Malawi, and South Africa. Due to the low sample size and limited distances explored the association between migration and distance is not robust or generalisable. In none of the models was the population size of the origin significantly associated with the migration parameter estimate (P=0.232; AIC = 179.16) (Supplementary Fig. S18). To be clear this applies specifically to these four countries and is an oversimplification of reality due to the discrete number of distances between the four countries explored in these migration models. However, in summary, the raw migration parameter estimates are negatively correlated with an increasing destination population size. This is sensible when placed alongside directional probability estimates whereby the higher population size is more likely to be the source of migration. Taken together for these GPSCs migrating between South Africa, Malawi, The Gambia, and Kenya this implies that the most migration is from large origin population sizes to smaller destinations.


(6) glm(θ∼α)

(7) glm(θ∼β)

(8) glm(θ∼γ)

### Estimating the weight of migration across four countries

#### Overview

We estimated symmetric migration parameters between four demes (6 migration parameters) (Supplementary Fig. S16). We expanded our prior to estimate parameters between the bound of 0 and 5 in line with the prior predictive analysis (Supplementary Fig. S3).

#### Weights

**Fig. 4.**
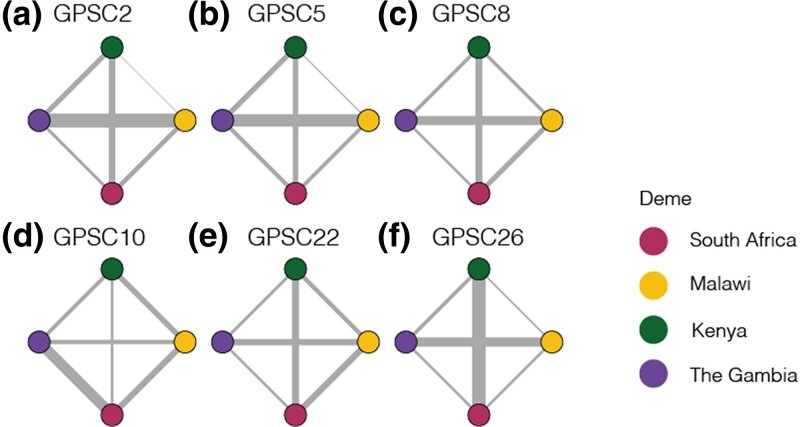
Migration parameter estimates for each GPSC. Migration paths weighted by the relative migration rate for each deme pair within each GPSC. The nodes are colored as follows: South Africa is represented in pink, Malawi in yellow, Kenya in Green, and The Gambia in purple.

To normalize the migration parameter estimates within each GPSCs, we repeated the same method described in Section “Weight.” For GPSC2 and GPSC5 migration between The Gambia and Malawi exceeded all other deme pairs in line with what was seen in the two-deme model. For GPSC8 the same pattern extended however migration between South Africa and Kenya followed closely behind. For GPSC26 migration between Kenya and South Africa was dominant with migration between The Gambia and Malawi being second highest. For GPSC10 migration between South Africa and the Gambia exceeded all other pairs and for GPSC22 migration between Kenya and The Gambia, and South Africa and The Gambia were lowest while all other deme pairs were similar (Fig. 4, Supplementary Table S2). In a generalized linear model, there is no association between the distance between demes (Equation 8). However, when comparing the relative population size between deme pairs to the migration parameter estimates there is a significant association (Coefficient =−0.027, P=0.00324; AIC = 101.13) in that as the relative population size increases between demes there is less migration between them.

### Between-country migration probability

**Fig. 5.**
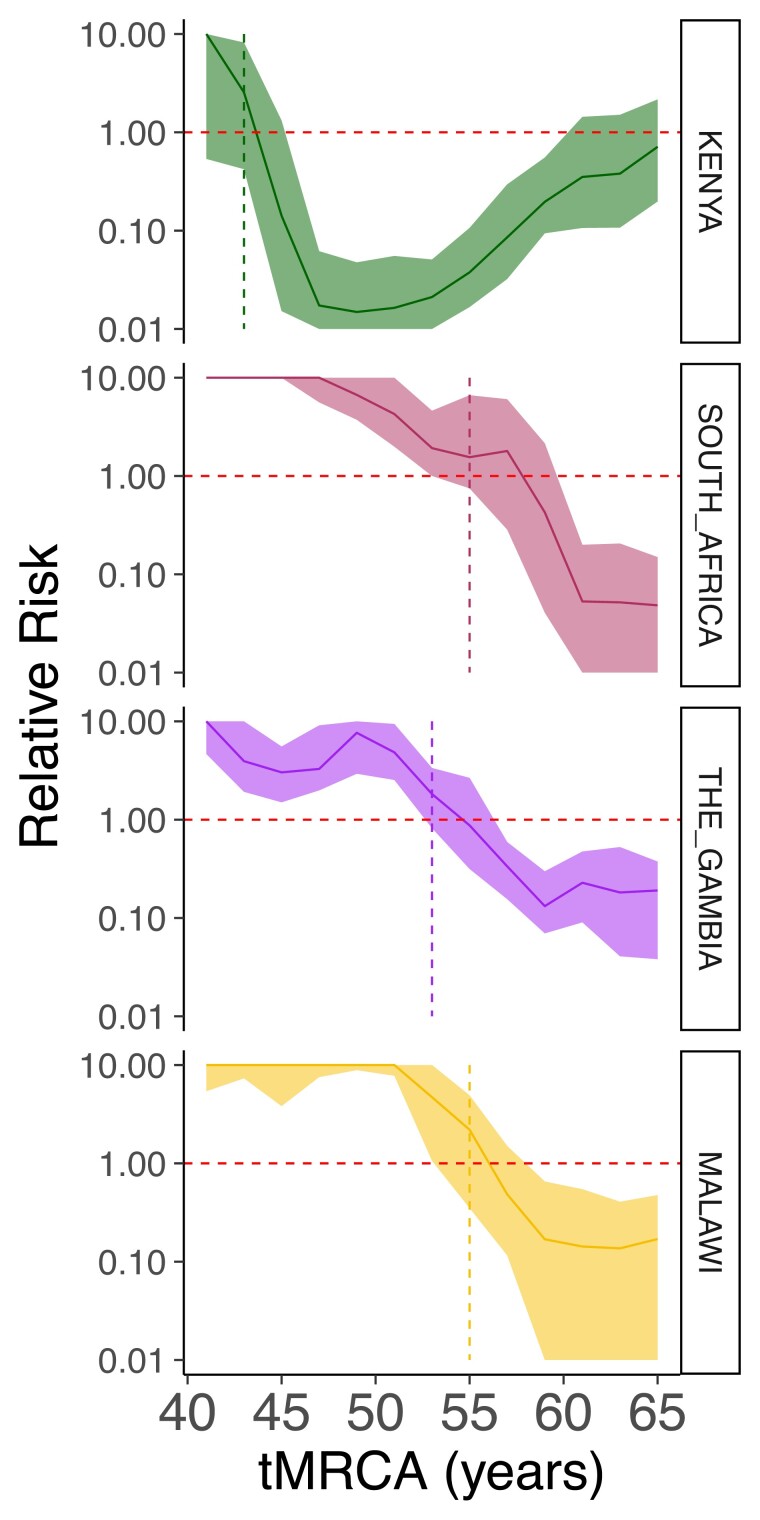
Out-of-country migration probability. The risk of pairs within divergence times across rolling 10-year time windows of being found within the same country as between each country and every other country for Kenya (green), South Africa (pink), The Gambia (purple), and Malawi (yellow). A dashed red line indicates a relative risk = 1 while a vertical dashed line for each plot denotes the tMRCA at which the relative risk crosses 1.

While here we are able to identify migration parameters when migration occurs we do not account for how often migration may occur but within the same country, or not occur at all. To address this, we used a risk ratio framework to investigate the risk of similarity across geographic distance (Salje *et al.* 2017; Lefrancq *et al.* 2022; Belman *et al.* 2023). We found that after 43 years of spread pairs are equally likely to be in Kenya as between Kenya and another country with an RR of 2.55 (95%CIs 0.42–8.19), for South Africa this is 55 years with an RR = 1.55 (95% CIs 0.75–6.63), for The Gambia it is after 53 years of spread; RR = 1.82 (95% CIs 0.82–3.36), and for Malawi this is after 55 years of spread; RR = 2.20 (95%CIs 0.35–4.92) (Fig. 5).

### Discussion

Our modeling framework utilizes genomic data and population summary statistics to infer migration parameters between demes that can best replicate the extant population distribution. We were able to successfully validate the method using simulated data both estimating the direction of migration between demes in the two-deme model, as well as the weight of migration across four demes. Interestingly, we detected some heterogeneity in migration rates across different lineages. The baseline expectation is that considerable variation would not exist between GPSCs. The similarity in migration between invasive and noninvasive lineages may imply, as found in Tonkin-Hill *et al.* (2022) that although the invasive lineages are not frequently found in carriage, they may persist at low population frequencies allowing them to be transmitted across borders in similar patterns (Supplementary Fig. S17).

We also find country-pair specific migration patterns which are in line with the source country usually being that with the greater population size when inferring directionality. However, when determining the demographic factor (population size of origin or destination and distance between them) with the greatest contribution to migration we found that the destination population size was more important.

Some limitations to this method include our inability to include the true carriage population sizes and true genome length within the simulation framework. As such, it is impossible to contextualize the migration parameters within the context of time, however, their relative relationship remains useful nevertheless. Further, as we use the population sizes for each deme in accordance with the true population sizes of those countries, some bias may be introduced since the pneumococcal carriage rate is known to vary by both country and human population. The age structure of each deme may consequently influence the true carriage rate in that a deme with a larger child population relative to the adult population may have more carriage overall, while simultaneously, a population with more children is unlikely to be as mobile as an adult population. Resolving this would require more demographic interrogation of pneumococcal carriage and human mobility in these regions. Our framework could also be applied on a smaller spatial scale where more granular data exists about the underlying carriage rates, and migration between regions, provinces, or states could consequently be inferred with better precision. Additionally, the ability for the migrating bacteria to take hold in a country depends on previous pathogen spread as well as vaccine campaign implementation.

Future implementations of our models could for example include parameter estimates for the population size of each deme, and incorporation of vaccine coverage and future immunity. Alternatively, the migration parameters could be incorporated into independent migration simulation frameworks which account for population immunity and other covariates. Human mobility data could in theory be used but currently (September 2023) representative between-country human-mobility data remains sparse. Meta provides travel data between-countries but these are dominated by high-income countries. Of the four demes included in our framework only South Africa is present within the Meta datasets from 2021 January to 2023 April. Interrogating openflights (https://openflights.org/data.html) between-country data for these four countries only provides sparse data for Kenya and Malawi, and no data for The Gambia. The majority of flights within Africa are not taken directly, they often include many stopovers which would further complicate the use of such data. Further, flight data would only represent a small subset of possible movements and in low-income countries it is largely reflecting tourism, while not accounting for the movement of the majority of the population (Findlater and Bogoch 2018; Gössling and Humpe 2020).

Estimates of migration rates such as those produced as part of this study could be informative for vaccine implementation policy. If there is more migration from deme A to B, implementing the vaccine first in deme A may have spillover effects into deme B, allowing such interventions to have an effect beyond country borders. Some possibilities for further development of our framework include incorporating additional countries, and inferring correlation between GPSC-specific migration patterns and classes of mobility such as flights compared to roads; or adults compared to children. This will help us to understand what types of mobility best explain the estimated migration parameters. Provided rich data from genomics-based surveillance systems, the current approach would also be applicable to multiple other species of bacteria.

## Supplementary Material

jkae058_Supplementary_Data

## Data Availability

The code and data associated with this paper are available at https://github.com/sophbel/LFI_between_country_migration/tree/main. Genomes are available on ENA and the Monocle database https://data.monocle.sanger.ac.uk/. Accession numbers are included in the additional data file “ERRs.csv” in the Github repository. Supplemental material available at G3 online.
