# Supplementary material for: Estimating between-country migration in pneumococcal populations
Source: G3 (Bethesda). 2024 Mar 20;14(6):jkae058. doi: 10.1093/g3journal/jkae058 (PMC11152062; doi:10.1093/g3journal/jkae058)
Supplement: jkae058_Supplementary_Data [file jkae058_supplementary_data.pdf]

Supplementary Material: Estimating Between Country Migration in Pneumococcal Populations

Sophie Belman[1], Henri Pesonen[2], Nicholas J. Croucher[5], Stephen D. Bentley[1†] and Jukka Corander[1,3,4†]

[1]Parasites and Microbes, Wellcome Sanger Institute, Hinxton, Cambridgeshire, UK, CB10 1SA

[2]Oslo Centre for Biostatistics and Epidemiology, Oslo University Hospital, Oslo, Norway, 0372

[3]Department of Biostatistics, University of Oslo, Oslo, Norway, 0371

[4]Helsinki Institute for Information Technology HIIT, Department of Mathematics and Statistics, University of Helsinki, Helsinki, Finland, 02150

[5]MRC Centre for Global Infectious Disease Analysis, Department of Infectious Disease Epidemiology, School of Public Health, White City Campus, Imperial College London, London, UK, W12 0BZ

[†]Co-senior authors

Supplementary Material: Estimating Between Country Migration in Pneumococcal Populations

Supplementary Figures

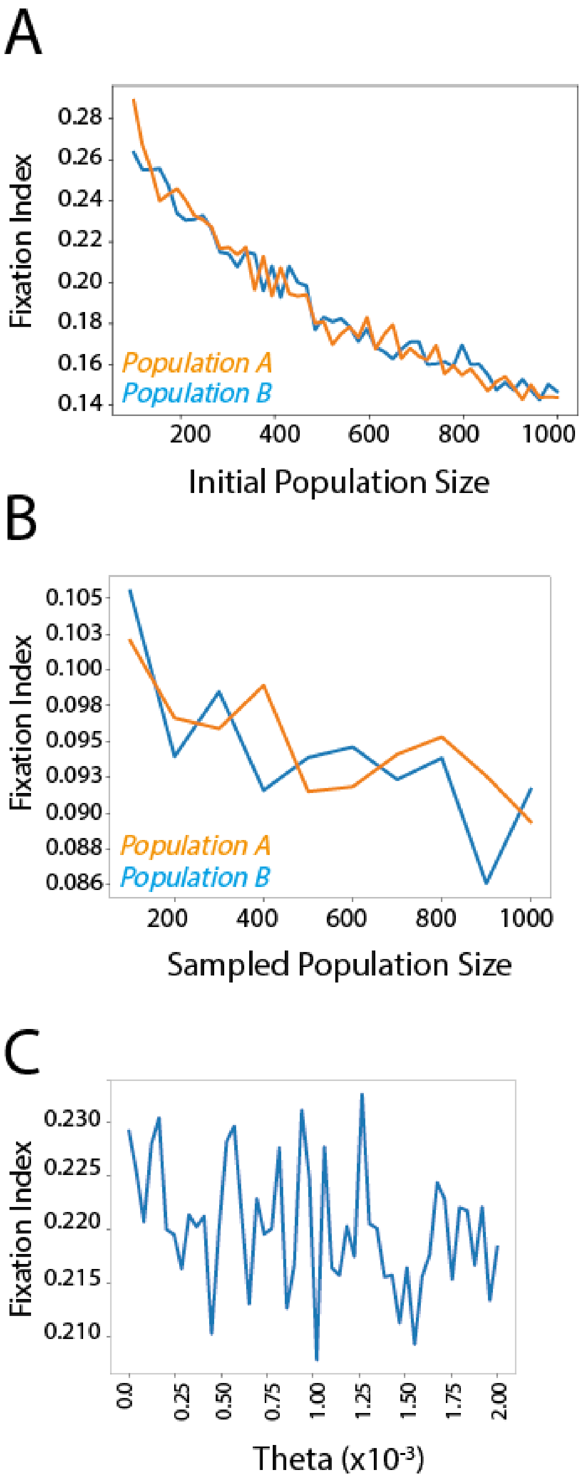

Figure S1 Sensitivity of the F_{st} value to simulation parameters fixed at a sequence length of 100. A) Initial population size fixing the alternate population size at 500 and the migration parameter at 2, sampling 100 from each. B) Sampled population size with a fixed migration parameter of 2, the sample size for the alternate population fixed at 500 and initial population parameters for A and B at 600 and 200 respectively. C) θ parameter (mutation rate) with a fixed migration parameter of 2, initial population sizes of A and B 600 and 200 respectively, sampling 100.

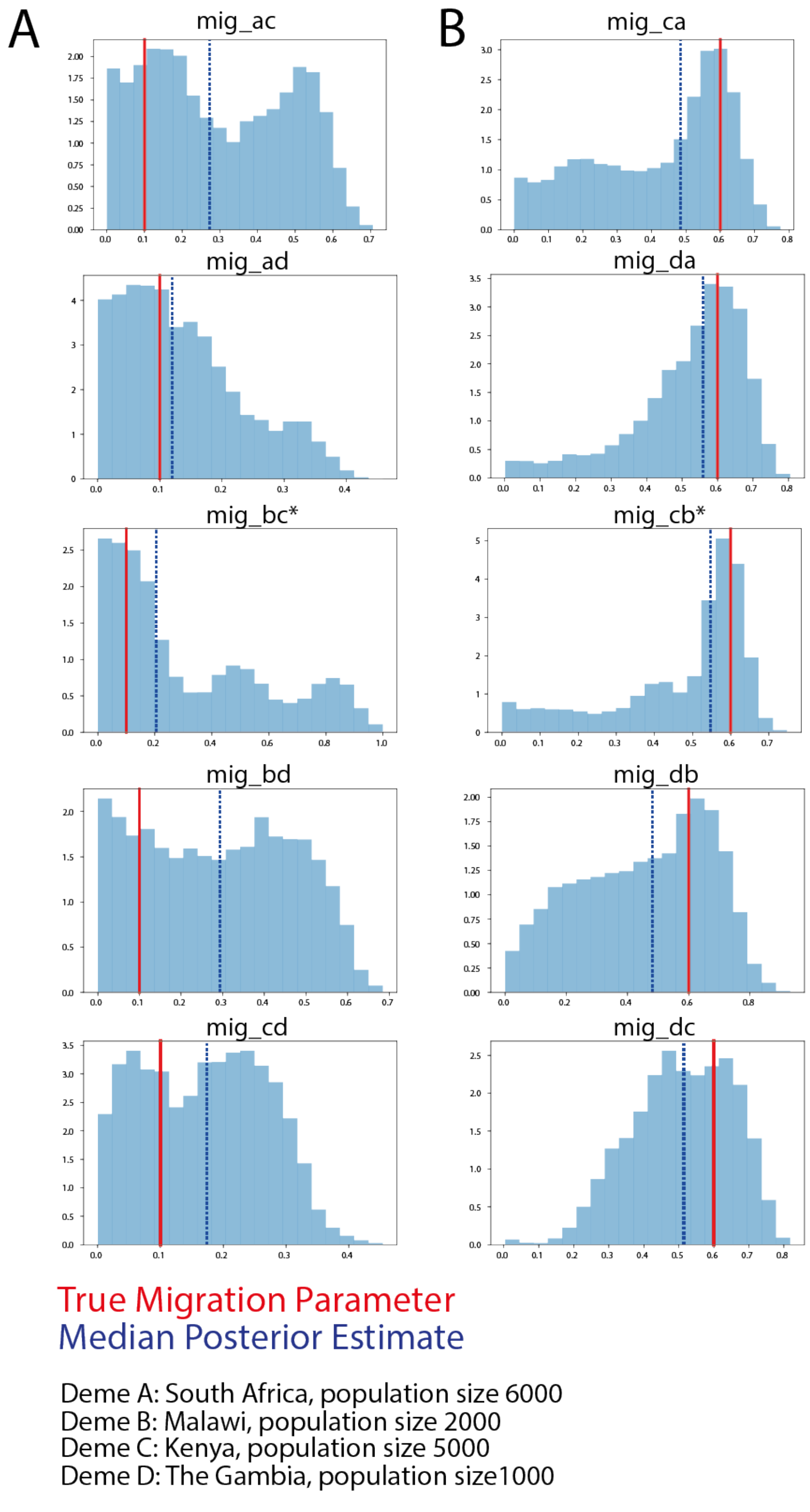

Figure S2 Recapturing input migration parameters with a 2 Deme model. A) The overlapping posterior density migration parameter estimates for migration parameter 1 — from a population $[a-d]$ to population $[a-d]$ B) and the inverse. The 'true' input parameters were mig_{a-d}=0.1 and mig_{a-d}=0.6. The posterior densities were estimated with a uniform prior and are visualized independently for each parameter (light blue), the true input parameter is indicated by the red vertical line while the median posterior estimate is indicated by the blue dashed line. Deme A=South Africa, initial population size 6000; Deme B=Malawi, initial population size 2000; Deme C=Kenya, initial population size 500; and Deme D=The Gambia, initial population size 1000.
*Used no-uturn (nuts) sampling rather than metropolis sampling for mig_bc and mig_cb due to difficulty converging.

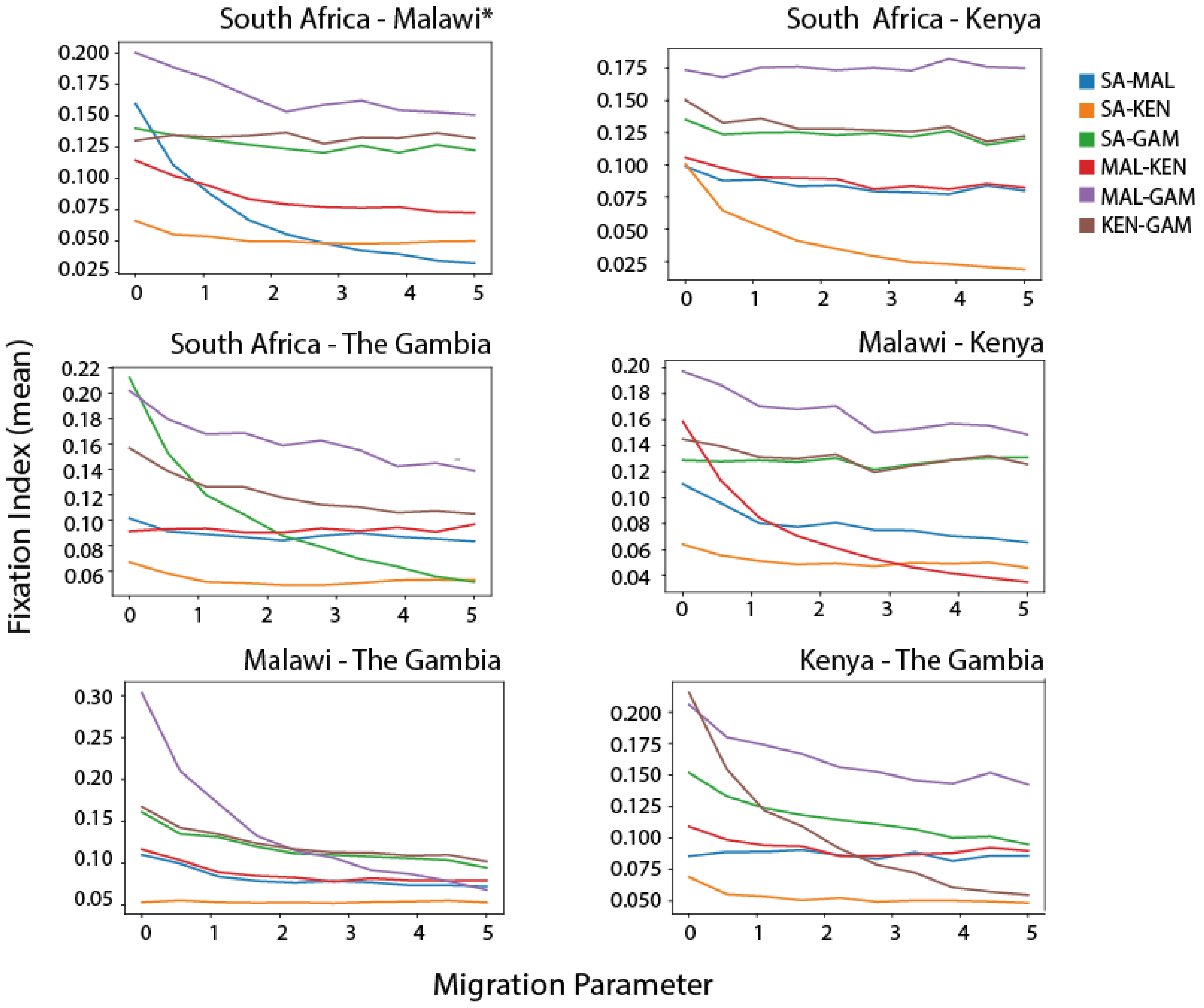

*Titled by the parameter which was adjusted in each plot

Figure S3 The response of the fixation index to varied migration parameters. Each plot indicates which migration parameter We varied and the F_{St} between the countries for each of those migration parameters is indicated by the colored lines.

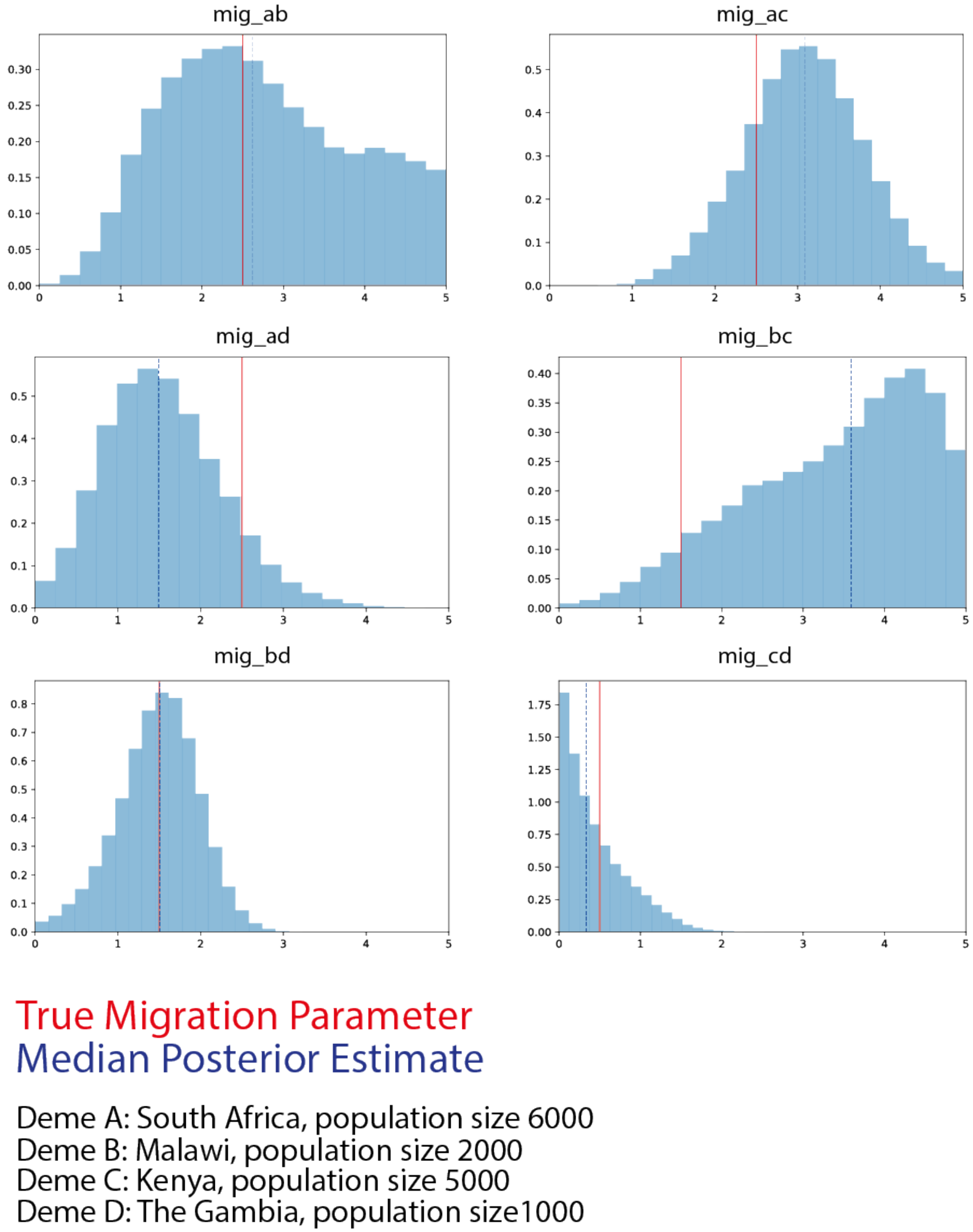

Figure S4 Recapturing migration parameters in the 4 deme model. The True Migration parameter is indicated by the red vertical line while the estimated median parameter is indicated by the blue-dashed vertical line. The posterior distribution density is represented by the blue histograms for each deme pair indicated by the title where a=South Africa, b=Malawi, c=Kenya, and d=The Gambia. The input population sizes for each of these scale to the true population size and are indicated in the figure.

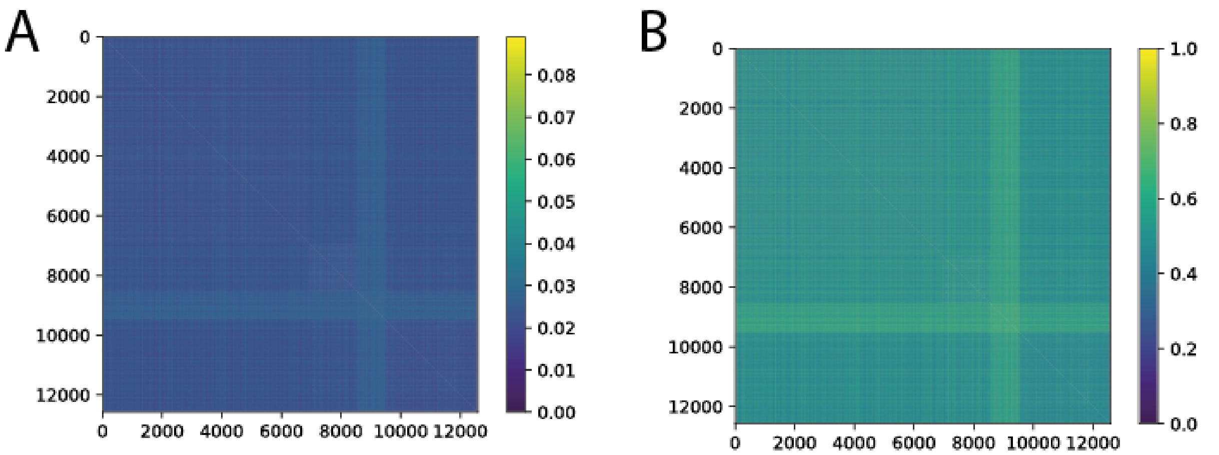

Figure S5 Pairwise distance estimates for between-country genomes across all 12,582 genome pairs from South Africa, Malawi, Kenya, and The Gambia, clustered in that order by A) Hamming distance and B) Jaccard Distance.

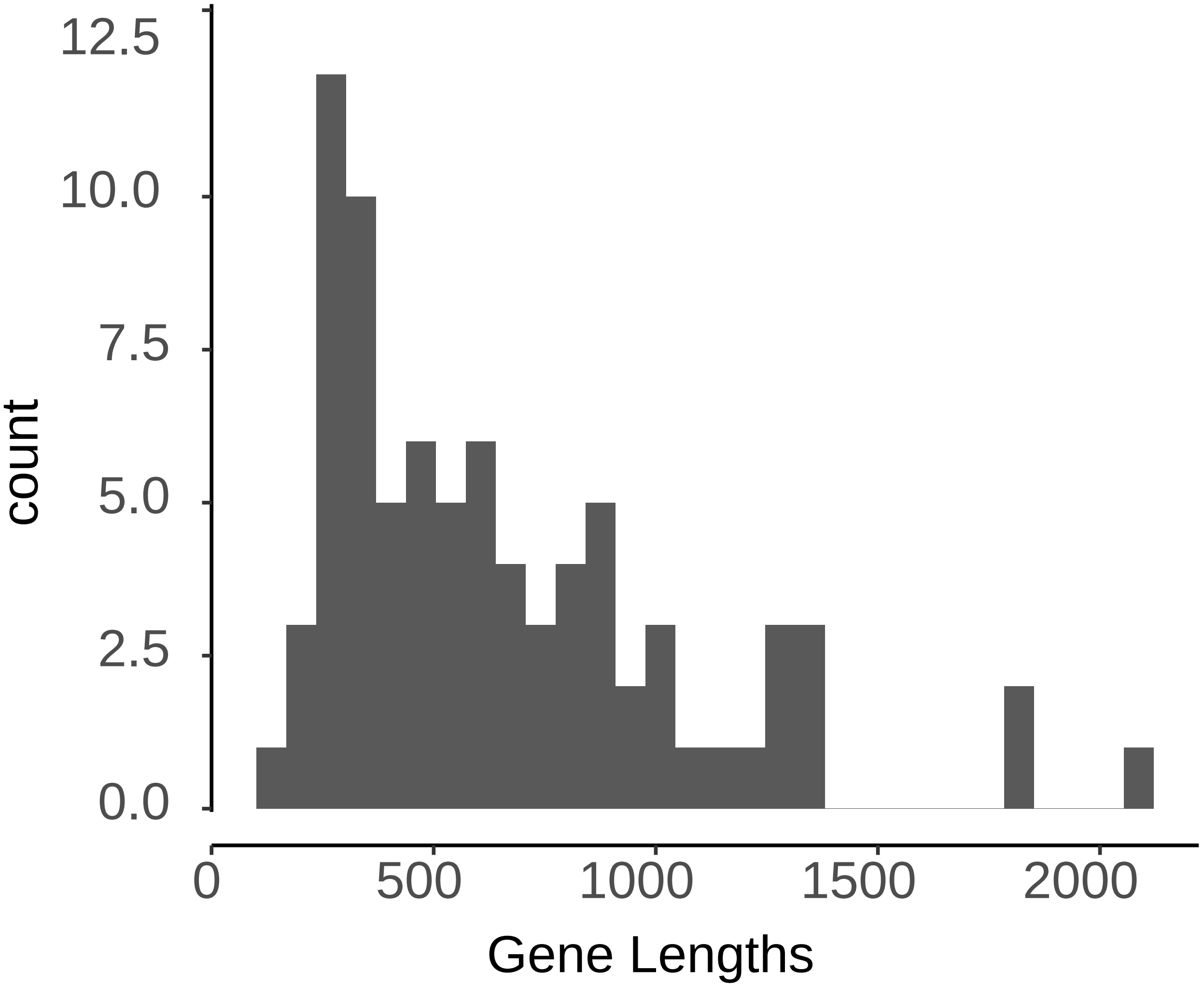

Figure S6 Histogram of gene length for each of the 81 neutral genes. Gene length is along the x-axis and the count is along the y-axis.

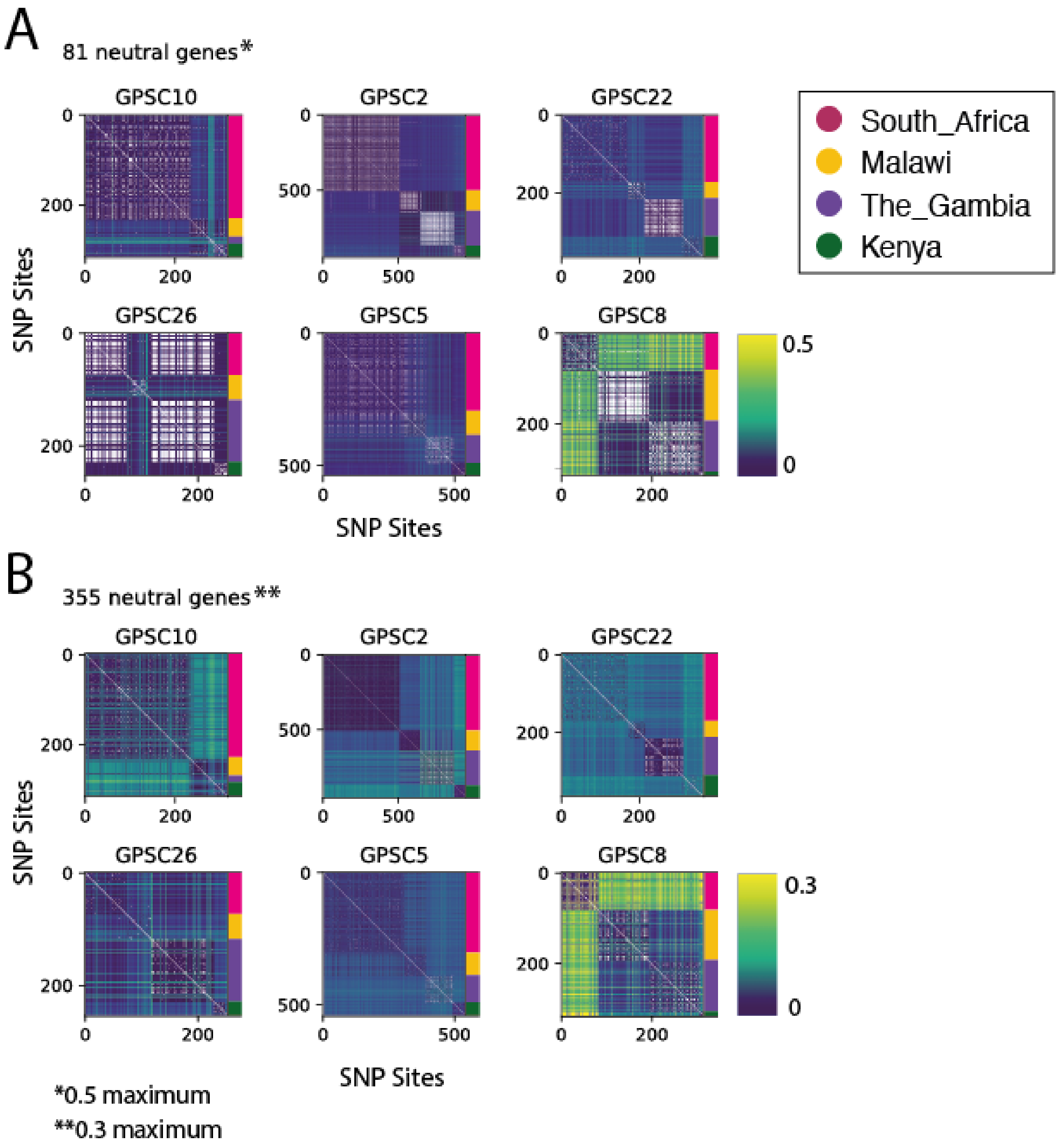

Figure S7 Pairwise Hamming distances across all genomes from each of the four demes (organized in the order of South Africa, Malawi, The Gambia, Kenya) for each GPSC in turn.These only include biallelic SNP sites. A) Includes 81 'neutral' genes. B)Includes 355 'neutral' genes.

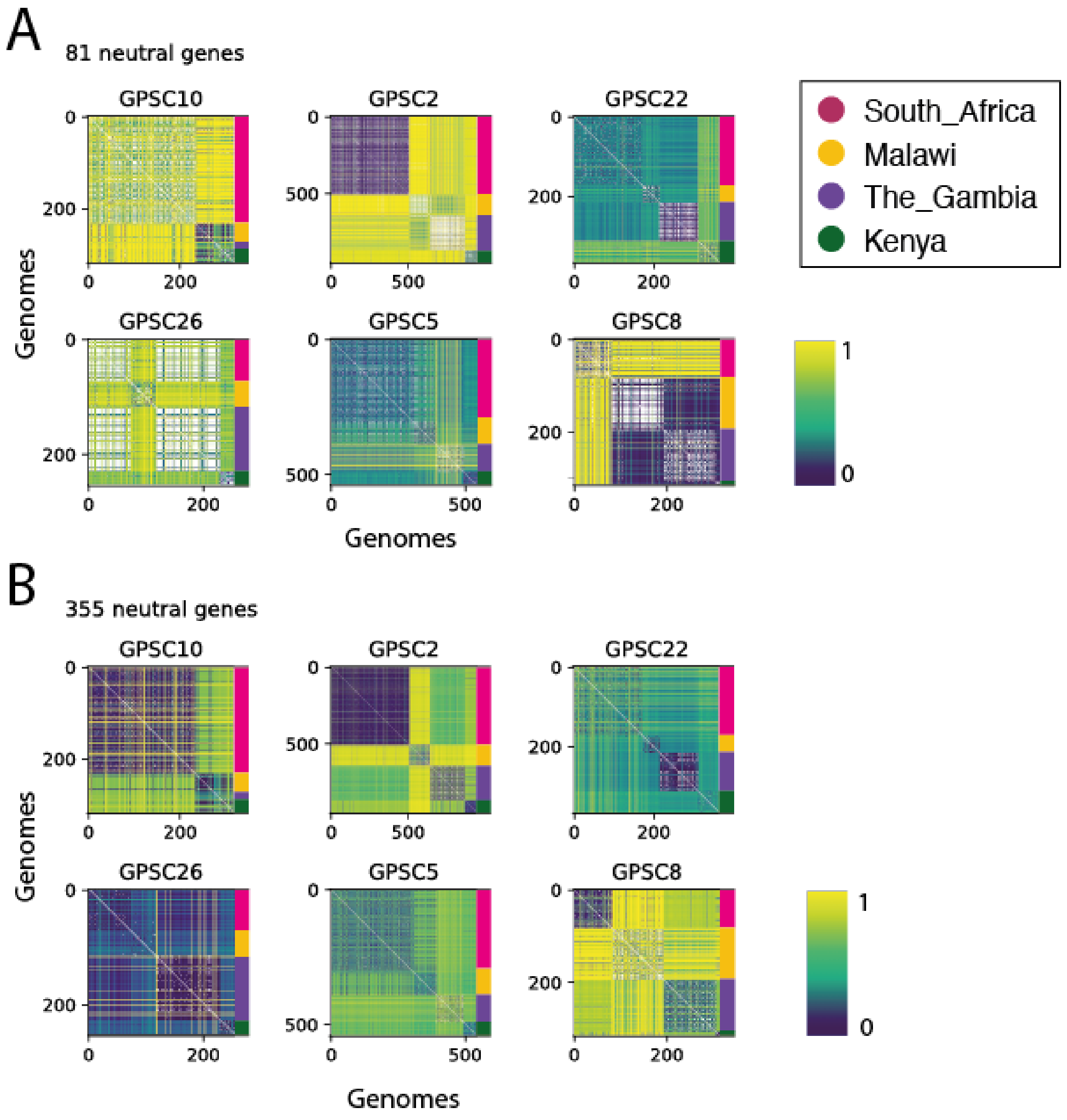

Figure S8 Pairwise Jaccard distances across all genomes from each of the four demes (organized in the order of South Africa, Malawi, The Gambia, Kenya) for each GPSC in turn. A) includes 81 'neutral' genes, B) includes 355 'neutral' genes. These only include biallelic SNP sites.

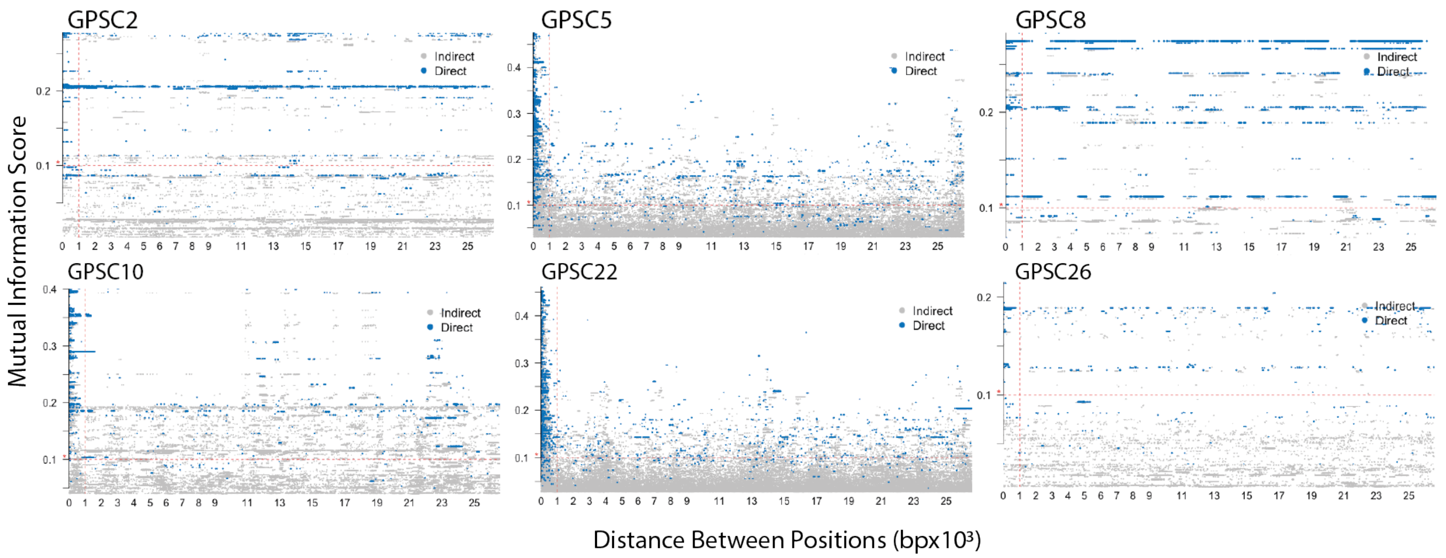

Figure S9 Mutual Information Scores between SNP pairs across 81 'neutral' gene alignments for each of the GPSCs. The vertical dashed line indicates the 1kb cutoff under which removed correlated sites. The horizontal dashed line indicates the 0.2 mutual information score cutoff which has been used previously for the pneumococcus.

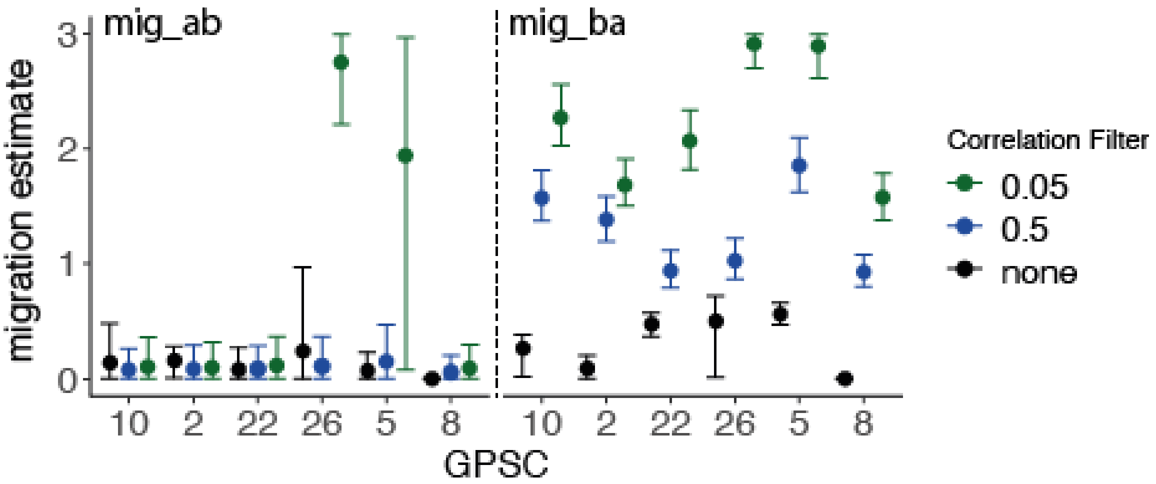

Figure S10 Estimated migration parameters removing correlated sites. Including mig_{ab} on the left and mig_{ba} on the right. Excluding all within a 1kb window upstream with r^2>0.5 (blue), and a r^2 > 0.05 (green), and retaining all sites (black). The error bars indicate 95% CIs and the x-axis indicates the GPSCs. Initial population sizes were for South Africa (deme A) and Malawi (deme B).

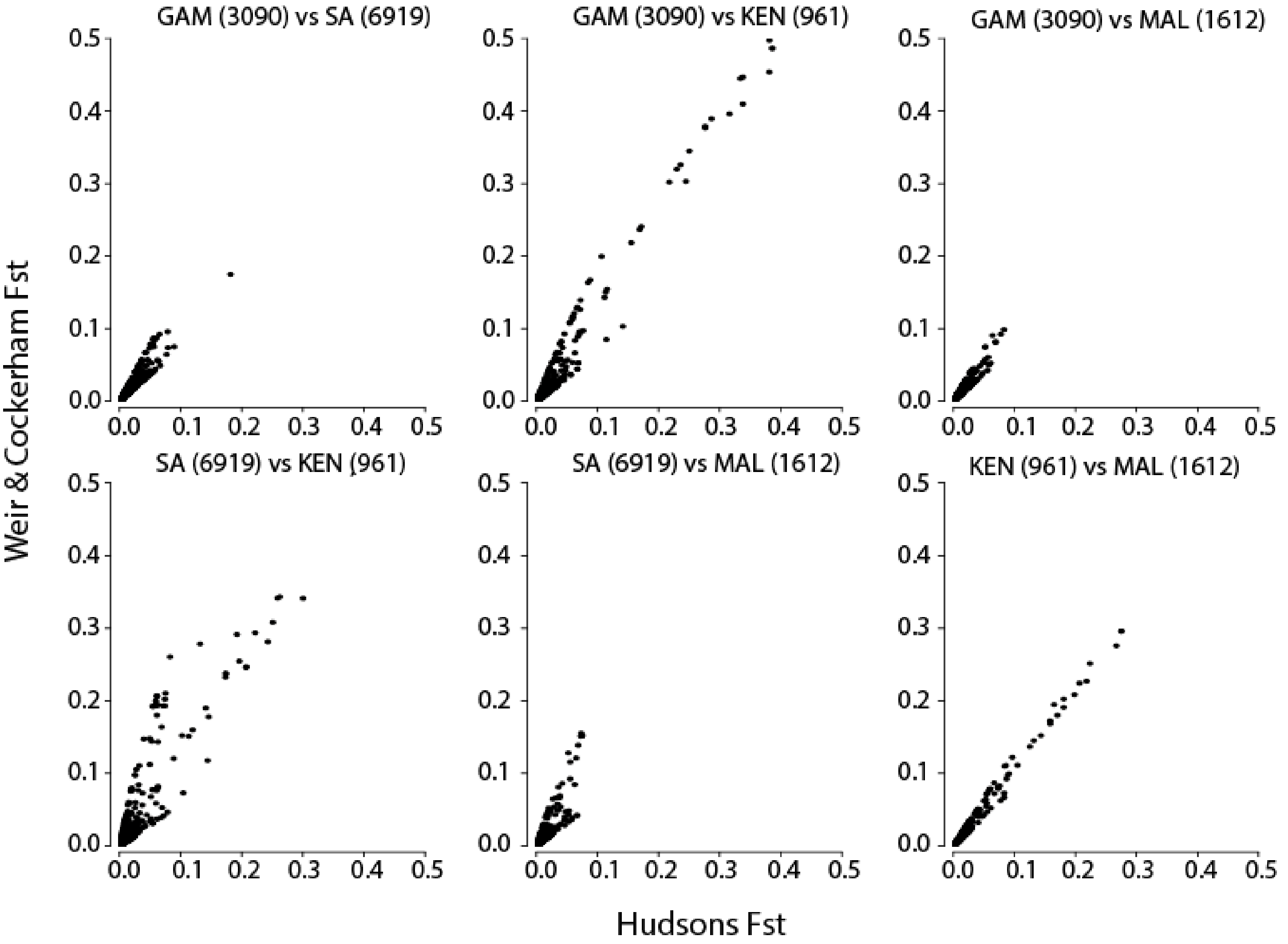

Figure S11 Pairwise comparison between the Hudson and Weir-Cockerham F_{st} values across all four demes. In total this includes six comparisons, one between each deme and every other deme.

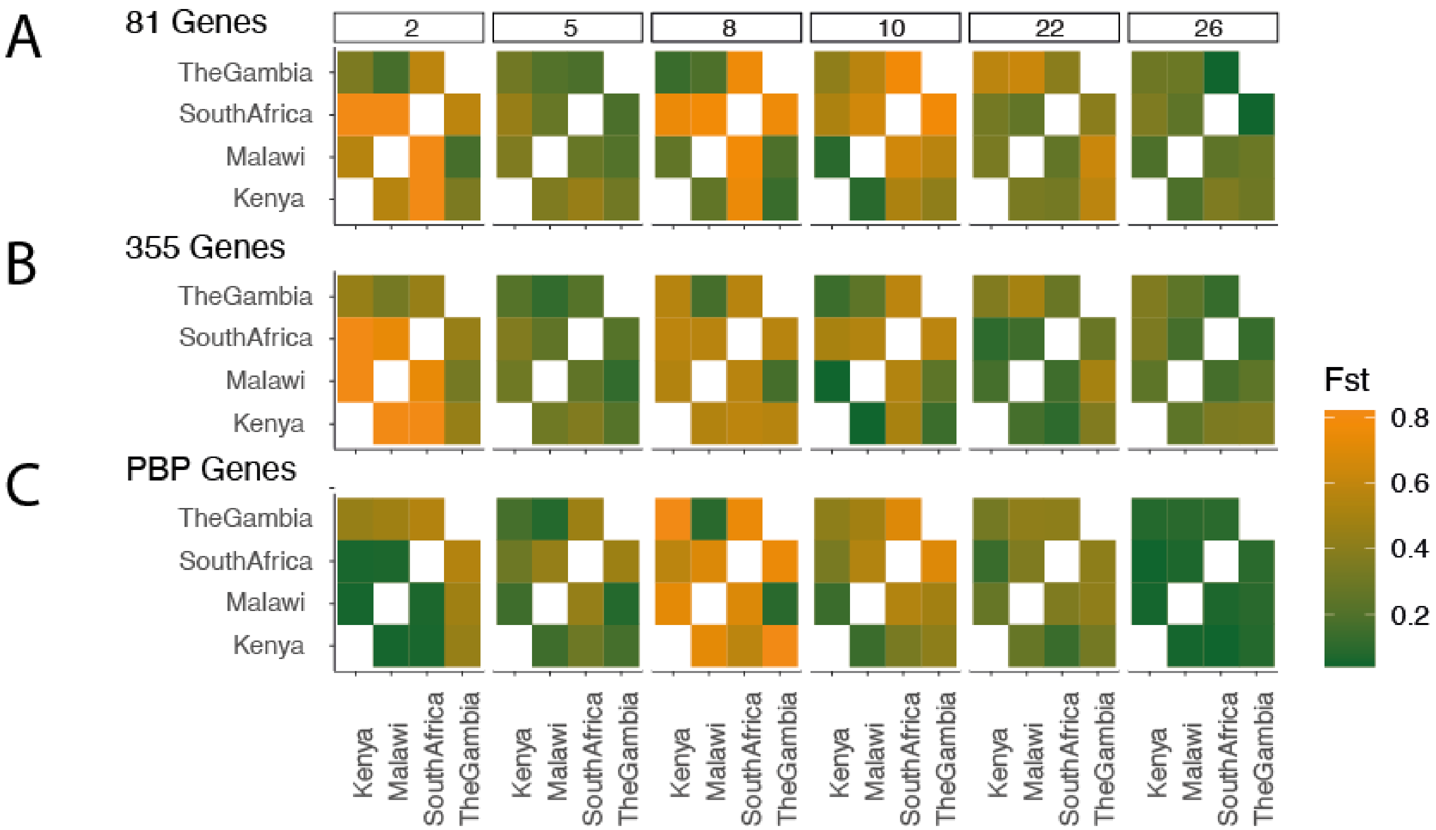

Figure S12 Hudsons F_{st} across all genomes between each of the four demes for each GPSC. A) calculated from 81 genes, B) from 355 genes, and C) only including the PBP genes (which are likely under selection in each place due to their interaction with penicillin-resistance acquisition). A higher F_{st} is a more divergent, separate population, while a lower F_{st} is a more highly mixing population, also known as panmictic.

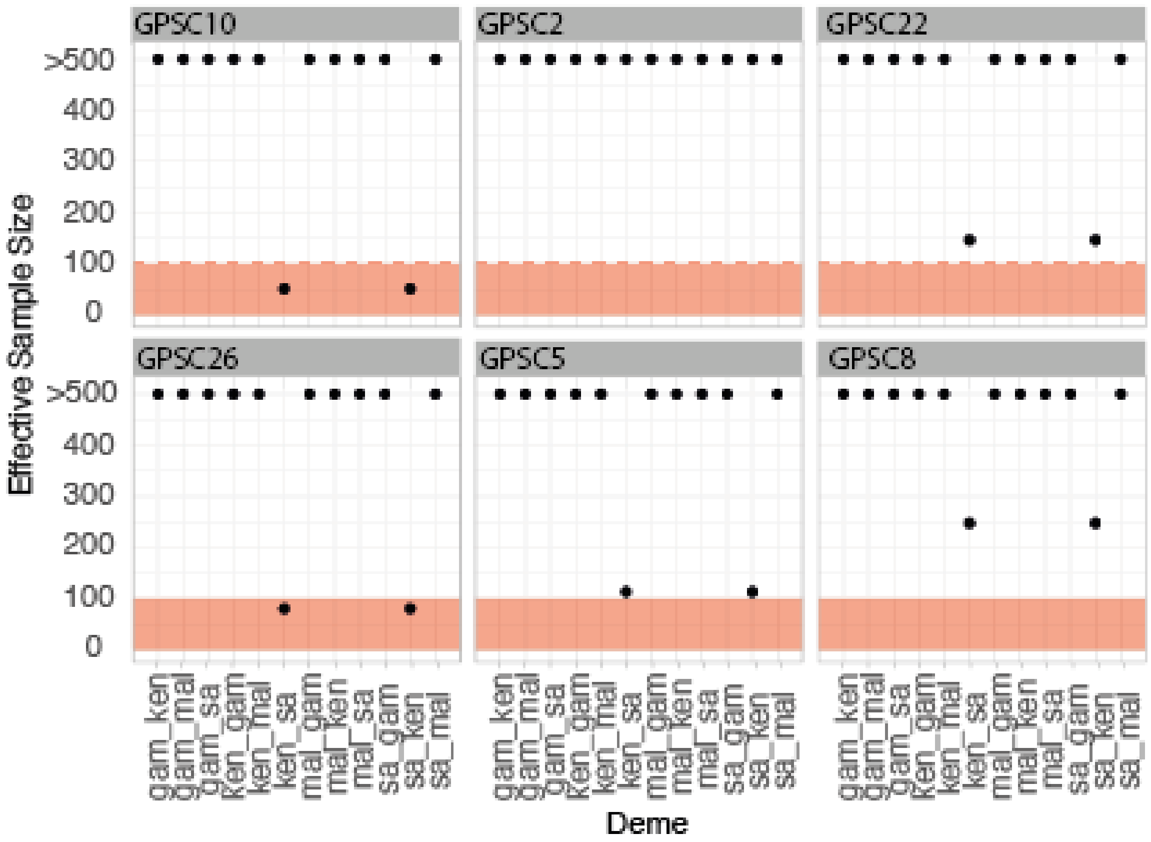

Figure S13 Convergence of asymmetric 2 deme parameter models. A) The effective sample size (ESS) across all parameters estimated. ESS <100 is indicated in red. B) The posterior density of parameter estimates between South Africa and Kenya for GPSC10. These were unable to converge due to the high co-linearity between them.

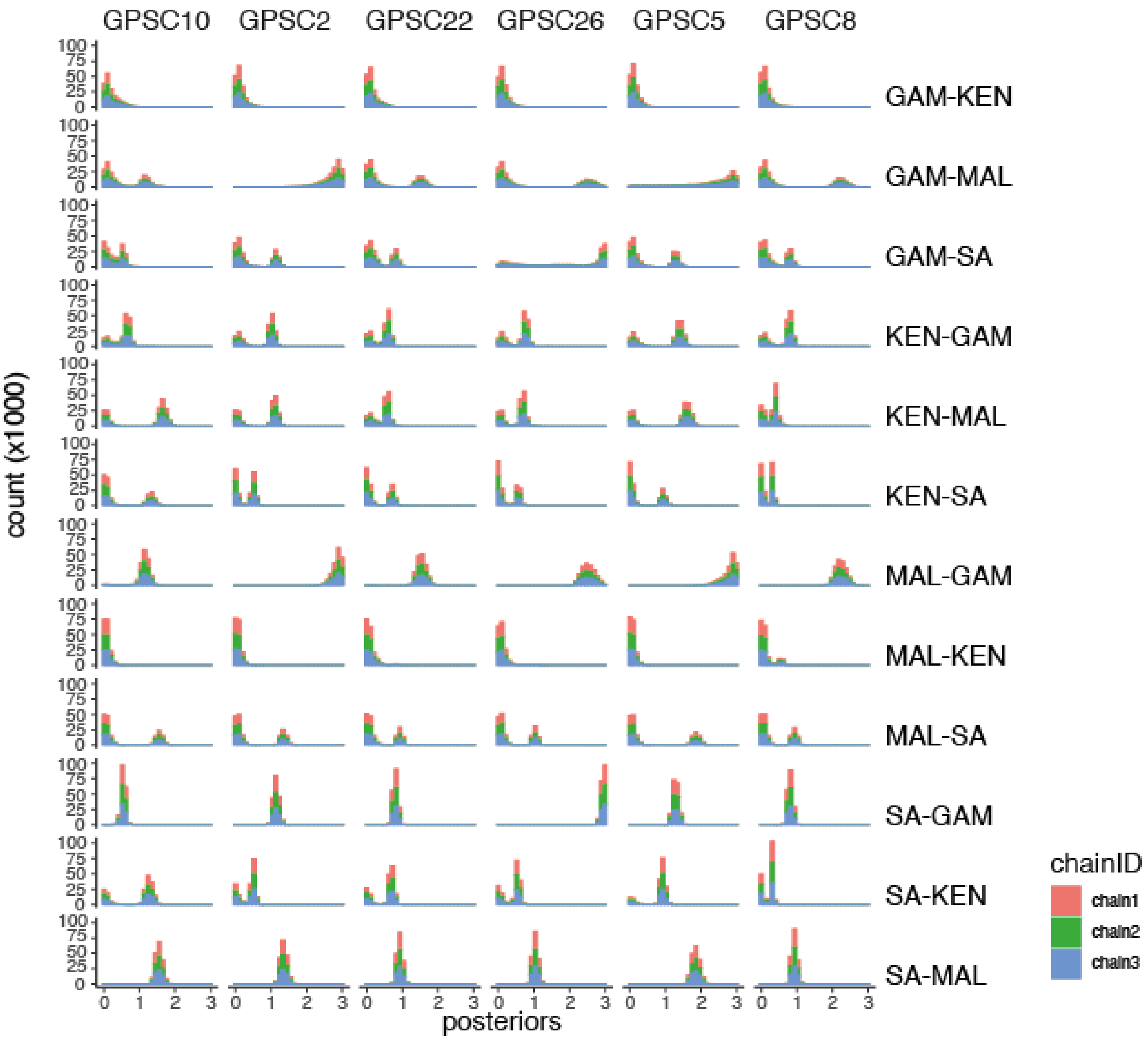

Figure S14 The posterior parameter distributions across 3 independent runs of 4 chains each across the 6 dominant GPSCs (columns) and 12 parameter estimates (rows).

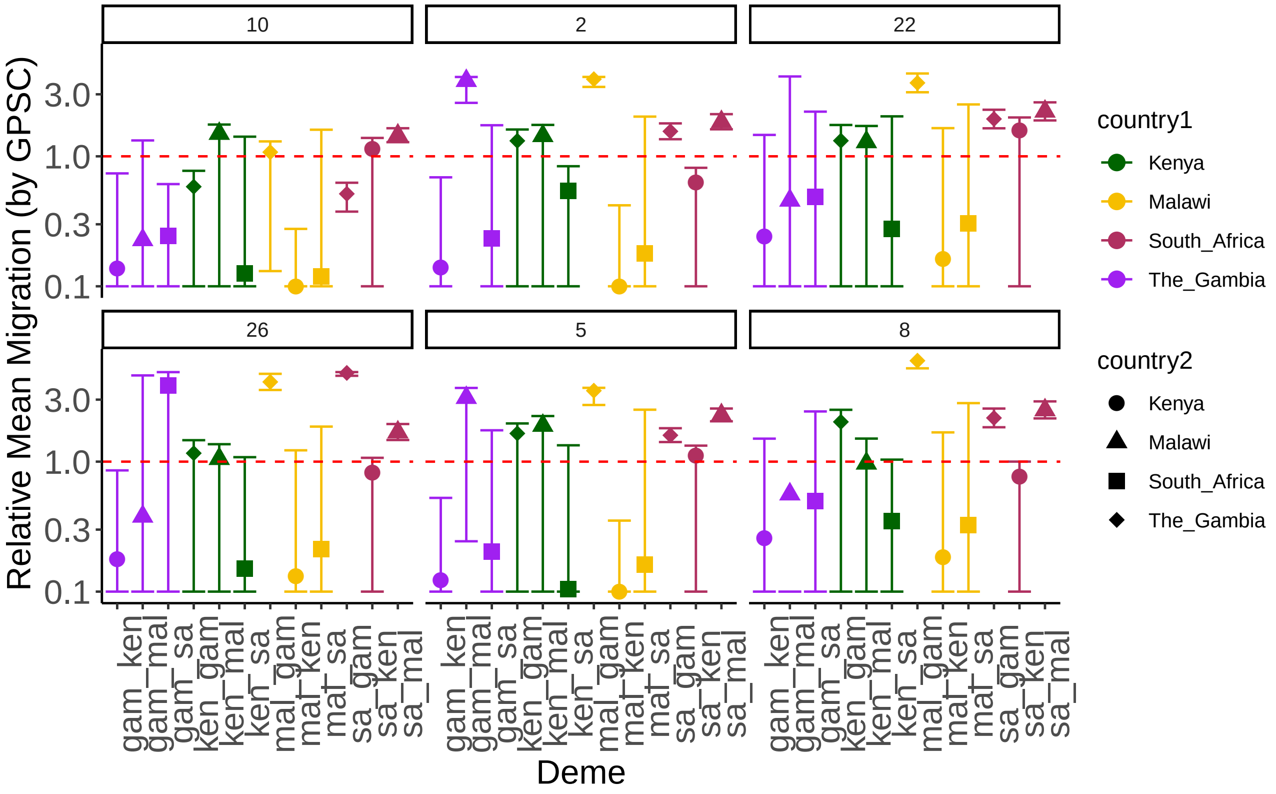

Figure S15 Relative migration parameters asymmetrically between two deme pairs. Relative migration for each deme pair within each GPSC independently. The x-axis indicated the deme and they are grouped by GPSC. The origin location of South Africa is represented in pink, Malawi in yellow, Kenya in Green, and The Gambia in purple.

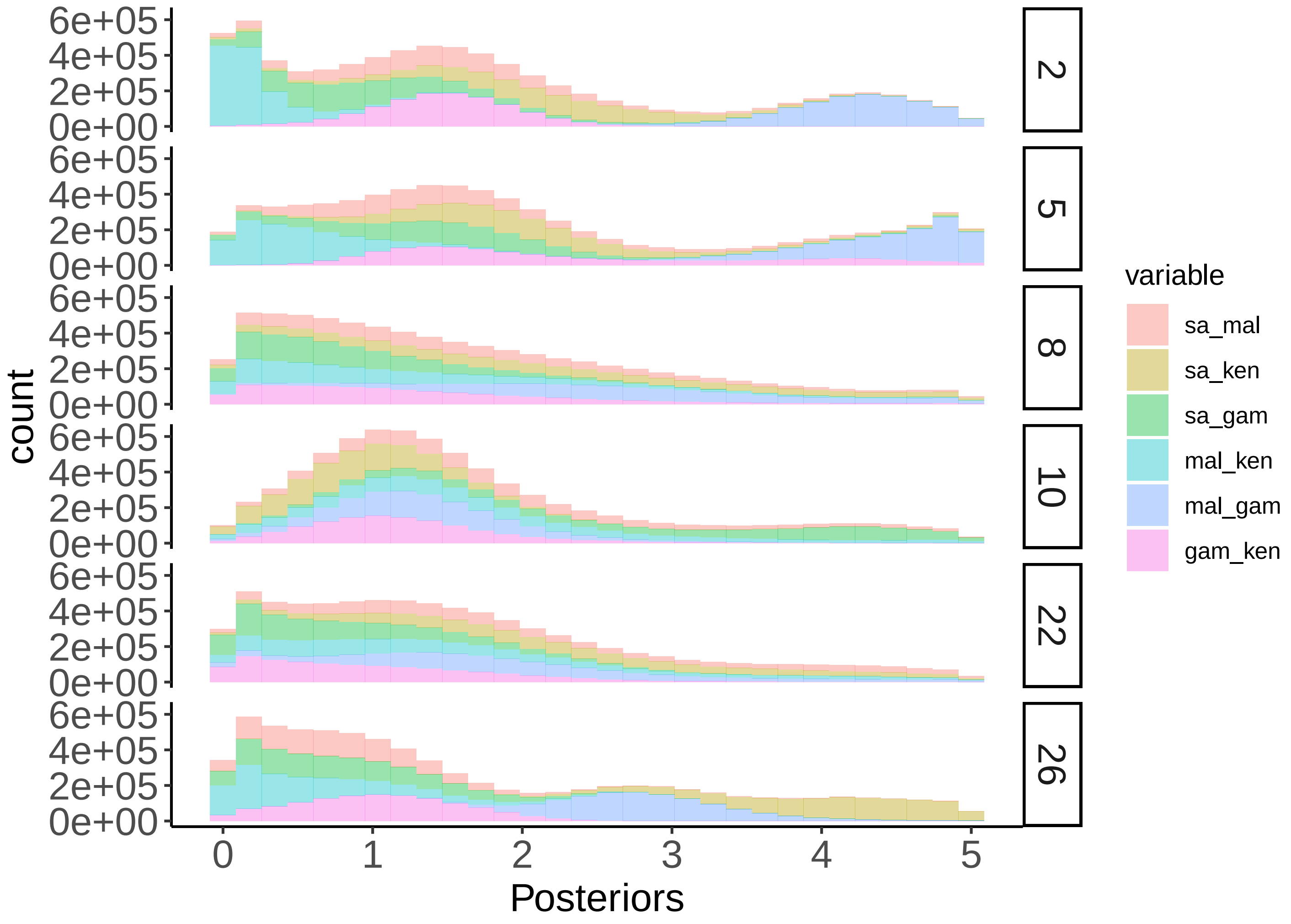

Figure S16 Posterior distributions for 6 parameter estimates for each GPSC, colored by parameter.

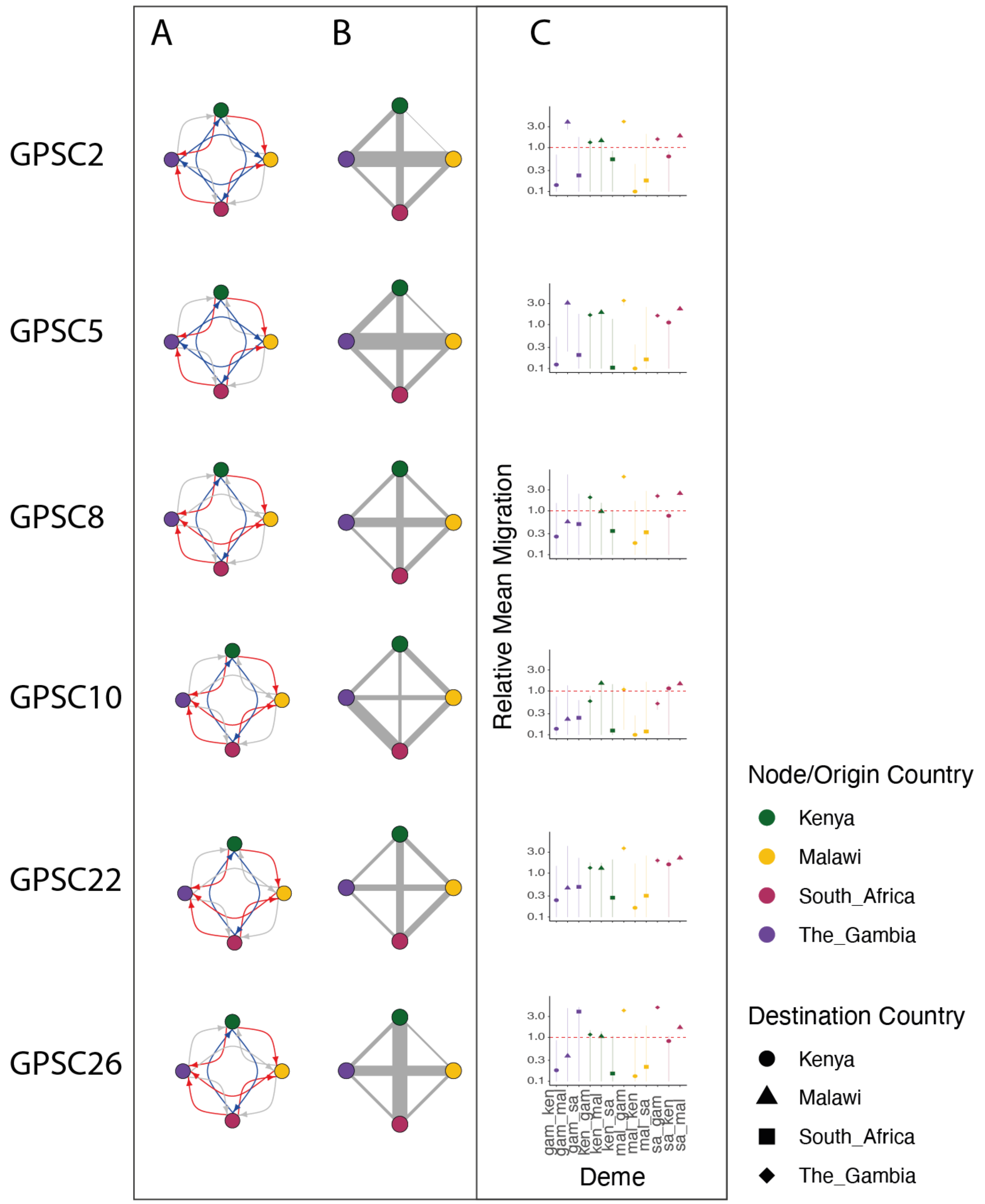

Figure S17 **Summary of each GPSC migration parameters** A) The directional probability from the 2 deme model for each GPSC whereby red= >0.6, blue = 0.4-0.6, and grey = 0.1-0.4 probability of migration asymmetrically for each deme pair. The Node colors are described in the legend. B) The weighted migration from the 4 deme model between all 4 demes. The node colors are the same as A. C) The rel-ative migration probability for each GPSC across all demes. The Origin country is colored the same as A and B and the Destination country is indicated in the legend.

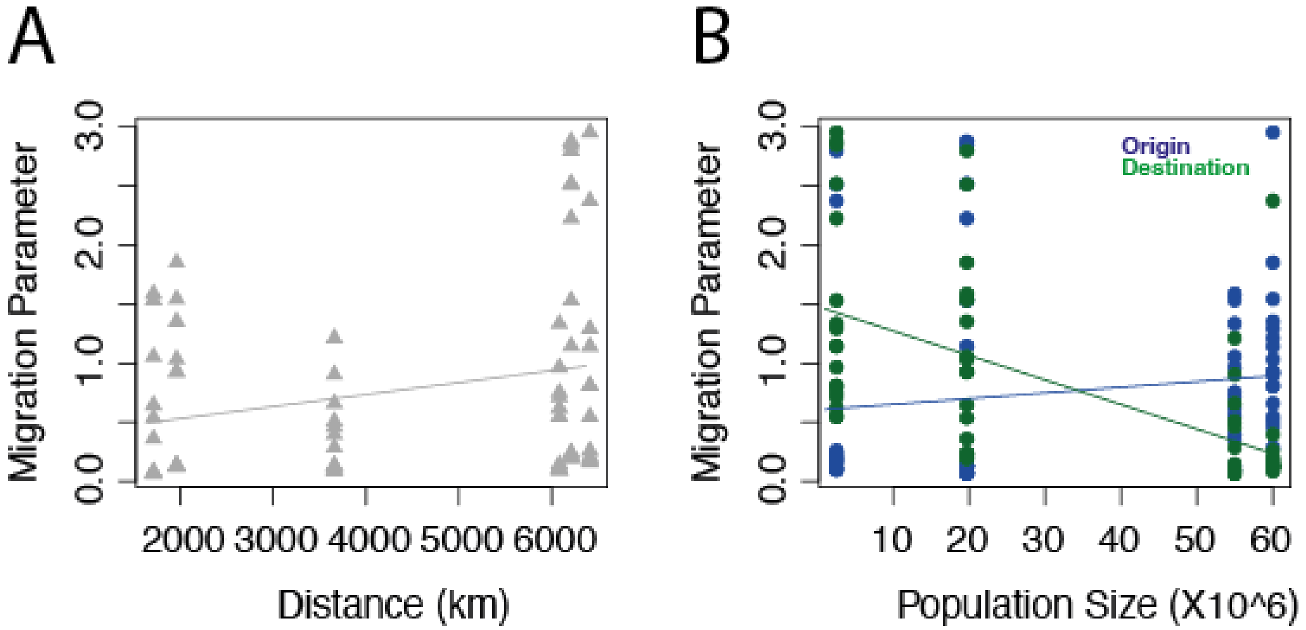

Figure S18 The population sizes and distance between countries versus migration parameter estimates. All plots include the migration parameter estimates (y-axis) against either distance between countries or the population size of the countries (x-axis). The left plot includes the distance between migration parameter demes (grey, triangles) and the right plot includes the population size of the origin (blue) or the destination (green). The models associated with each figure are included in lines of the same color.

Supplementary Tables

Table S1 Parameter estimates across all pairs within the two deme model. Values within the square brackets denote the 95% credible intervals. The 'Parameter' is the raw migration parameter estimate while the 'Relative Parameter' is relative to all other deme pairs *within* each GPSC. The 'Directional Migration Probability' is the probability of migration asymmetrically for each GPSC and each deme pair (ie for $sa - mal$, GPSC10 there is 0.667 probability of migration while for $mal - sa$ there is $(1 - 0.667)$ probability of migration.

Deme	GPSC	Parameter	Relative Parameter	Directional Migration Probability	ESS
sa_mal	10	1.542[1.365-1.747]	1.451[1.285-1.644]	0.667	13594.51
sa_mal	2	1.352[1.184-1.549]	1.843[1.614-2.111]	0.667	10837.93
sa_mal	22	0.923[0.787-1.084]	2.215[1.888-2.602]	0.666	12514.85
sa_mal	26	1.033[0.9-1.195]	1.683[1.467-1.947]	0.667	15763.81
sa_mal	5	1.851[1.655-2.075]	2.286[2.044-2.562]	0.667	11029.98
sa_mal	8	0.927[0.8-1.082]	2.492[2.151-2.908]	0.667	21707.4
sa_ken	10	1.211[0.007-1.472]	1.14[0.007-1.385]	0.507	50.63545
sa_ken	2	0.462[0.004-0.599]	0.629[0.006-0.816]	0.411	982.8784
sa_ken	22	0.659[0.006-0.829]	1.581[0.015-1.99]	0.496	145.9672
sa_ken	26	0.507[0.005-0.657]	0.827[0.008-1.07]	0.479	80.22054
sa_ken	5	0.902[0.016-1.075]	1.114[0.02-1.328]	0.577	113.1349
sa_ken	8	0.286[0.002-0.372]	0.77[0.005-1.001]	0.424	247.5898
sa_gam	10	0.547[0.399-0.665]	0.515[0.375-0.626]	0.627	6574.967
sa_gam	2	1.142[0.993-1.315]	1.557[1.354-1.792]	0.667	24110.21
sa_gam	22	0.807[0.684-0.951]	1.938[1.642-2.283]	0.665	23602.96
sa_gam	26	2.953[2.808-2.998]	4.814[4.578-4.887]	0.64	10354.19
sa_gam	5	1.292[1.146-1.464]	1.595[1.415-1.808]	0.667	21663.93
sa_gam	8	0.807[0.684-0.953]	2.17[1.839-2.561]	0.664	18415.62
mal_ken	10	0.066[0.003-0.294]	0.062[0.002-0.276]	0	12223.04
mal_ken	2	0.064[0.002-0.308]	0.088[0.003-0.42]	0	10179.53
mal_ken	22	0.068[0.002-0.686]	0.162[0.005-1.647]	0.024	615.344
mal_ken	26	0.08[0.003-0.75]	0.131[0.005-1.222]	0.021	1909.27
mal_ken	5	0.063[0.002-0.285]	0.077[0.003-0.352]	0	11555.99
mal_ken	8	0.069[0.003-0.624]	0.185[0.007-1.679]	0.097	826.4831
mal_gam	10	1.144[0.139-1.384]	1.077[0.131-1.302]	0.638	514.1442
mal_gam	2	2.878[2.51-2.995]	3.921[3.419-4.081]	0.454	6888.511
mal_gam	22	1.53[1.296-1.807]	3.672[3.111-4.338]	0.666	5317.546
mal_gam	26	2.518[2.184-2.907]	4.105[3.559-4.738]	0.667	6652.609
mal_gam	5	2.846[2.21-2.994]	3.515[2.729-3.698]	0.529	1395.229
mal_gam	8	2.224[1.943-2.565]	5.978[5.223-6.896]	0.667	9834.932
gam_ken	10	0.146[0.006-0.785]	0.137[0.005-0.739]	0.047	6380.046
gam_ken	2	0.103[0.004-0.506]	0.14[0.005-0.689]	0.002	9089.028
gam_ken	22	0.101[0.004-0.609]	0.241[0.009-1.461]	0.048	5777.879
gam_ken	26	0.109[0.004-0.526]	0.178[0.007-0.857]	0.011	8371.036
gam_ken	5	0.099[0.004-0.426]	0.123[0.005-0.526]	0	12688.59
gam_ken	8	0.096[0.003-0.559]	0.259[0.009-1.503]	0.005	5378.5
mal_sa	10	0.127[0.004-1.702]	0.119[0.004-1.602]	0.333	13594.51
mal_sa	2	0.131[0.004-1.483]	0.178[0.006-2.02]	0.333	10837.93
mal_sa	22	0.127[0.004-1.044]	0.305[0.009-2.507]	0.334	12514.85
mal_sa	26	0.131[0.004-1.141]	0.213[0.007-1.861]	0.333	15763.81
mal_sa	5	0.13[0.004-2.032]	0.161[0.005-2.509]	0.333	11029.98
mal_sa	8	0.121[0.004-1.049]	0.324[0.01-2.82]	0.333	21707.4
ken_sa	10	0.133[0.004-1.505]	0.125[0.003-1.416]	0.493	50.63545
ken_sa	2	0.397[0.002-0.616]	0.541[0.003-0.839]	0.589	982.8784
ken_sa	22	0.115[0.003-0.846]	0.275[0.006-2.03]	0.504	145.9672
ken_sa	26	0.092[0.002-0.664]	0.15[0.003-1.082]	0.521	80.22054
ken_sa	5	0.084[0.002-1.082]	0.104[0.003-1.336]	0.423	113.1349
ken_sa	8	0.13[0.002-0.385]	0.348[0.005-1.036]	0.576	247.5898
gam_sa	10	0.259[0.004-0.65]	0.244[0.004-0.611]	0.373	6574.967

Table S1 – continued from previous page

Deme	GPSC	Parameter	Relative Parameter	Directional Migration Probability	ESS
gam_sa	2	0.171[0.005-1.273]	0.233[0.007-1.734]	0.333	24110.21
gam_sa	22	0.203[0.006-0.919]	0.487[0.014-2.206]	0.335	23602.96
gam_sa	26	2.374[0.053-2.995]	3.869[0.086-4.882]	0.36	10354.19
gam_sa	5	0.164[0.005-1.411]	0.202[0.006-1.743]	0.333	21663.93
gam_sa	8	0.185[0.005-0.905]	0.498[0.013-2.434]	0.336	18415.62
ken_mal	10	1.588[0.008-1.865]	1.494[0.007-1.755]	1	12223.04
ken_mal	2	1.054[0.007-1.28]	1.436[0.01-1.744]	1	10179.53
ken_mal	22	0.537[0.012-0.713]	1.289[0.028-1.712]	0.976	615.344
ken_mal	26	0.645[0.009-0.835]	1.052[0.014-1.362]	0.979	1909.27
ken_mal	5	1.534[0.008-1.816]	1.894[0.01-2.242]	1	11555.99
ken_mal	8	0.361[0.005-0.56]	0.969[0.013-1.505]	0.903	826.4831
gam_mal	10	0.241[0.007-1.408]	0.227[0.007-1.325]	0.362	514.1442
gam_mal	2	2.797[1.892-2.993]	3.812[2.577-4.077]	0.546	6888.511
gam_mal	22	0.191[0.006-1.714]	0.457[0.013-4.114]	0.334	5317.546
gam_mal	26	0.232[0.007-2.826]	0.379[0.011-4.606]	0.333	6652.609
gam_mal	5	2.512[0.198-2.988]	3.102[0.244-3.69]	0.471	1395.229
gam_mal	8	0.209[0.007-2.484]	0.562[0.017-6.679]	0.333	9834.932
ken_gam	10	0.62[0.015-0.822]	0.584[0.014-0.774]	0.953	6380.046
ken_gam	2	0.966[0.012-1.18]	1.316[0.017-1.608]	0.998	9089.028
ken_gam	22	0.549[0.011-0.726]	1.319[0.027-1.742]	0.952	5777.879
ken_gam	26	0.712[0.018-0.897]	1.161[0.03-1.462]	0.989	8371.036
ken_gam	5	1.335[0.016-1.593]	1.648[0.019-1.968]	1	12688.59
ken_gam	8	0.753[0.012-0.932]	2.023[0.031-2.506]	0.995	5378.5

Table S2 Migration parameter estimates symmetrically across four demes for six GPSCs. Values within the square brackets denote the 95% credible intervals. The 'Parameter' is the raw migration parameter estimate while the 'Relative Parameter' is relative to all other deme pairs *within* each GPSC.

Deme	GPSC	Parameter	Relative Parameter
sa_mal	10	1.761[0.241-4.655]	1.031[0.141-2.725]
sa_ken	10	0.841[0.064-1.916]	0.492[0.037-1.121]
sa_gam	10	3.156[0.57-4.862]	1.847[0.333-2.846]
mal_ken	10	1.606[0.106-4.751]	0.94[0.062-2.781]
mal_gam	10	1.336[0.253-2.863]	0.782[0.148-1.676]
gam_ken	10	1.119[0.186-2.847]	0.655[0.109-1.666]
sa_mal	2	1.465[0.108-3.99]	0.833[0.061-2.268]
sa_ken	2	2.135[0.267-3.816]	1.213[0.152-2.169]
sa_gam	2	0.886[0.076-2.675]	0.504[0.043-1.52]
mal_ken	2	0.138[0.005-0.961]	0.078[0.003-0.546]
mal_gam	2	4.242[3.047-4.937]	2.411[1.732-2.806]
gam_ken	2	1.476[0.462-2.383]	0.839[0.262-1.354]
sa_mal	22	1.755[0.121-4.77]	1.077[0.074-2.927]
sa_ken	22	2.111[0.204-4.721]	1.295[0.125-2.897]
sa_gam	22	0.726[0.023-2.728]	0.445[0.014-1.674]
mal_ken	22	1.258[0.063-4.492]	0.772[0.039-2.757]
mal_gam	22	1.777[0.109-4.608]	1.091[0.067-2.828]
gam_ken	22	0.863[0.03-3.116]	0.53[0.019-1.912]
sa_mal	26	0.805[0.042-3.071]	0.473[0.025-1.802]
sa_ken	26	4.07[2.368-4.948]	2.388[1.39-2.903]
sa_gam	26	0.779[0.032-2.133]	0.457[0.019-1.251]
mal_ken	26	0.464[0.015-2.029]	0.272[0.009-1.191]
mal_gam	26	2.777[1.818-4.105]	1.63[1.067-2.409]
gam_ken	26	1.027[0.082-2.095]	0.602[0.048-1.229]
sa_mal	5	1.42[0.178-4.208]	0.684[0.086-2.028]
sa_ken	5	1.935[0.679-4.668]	0.933[0.327-2.25]
sa_gam	5	1.45[0.088-4.149]	0.699[0.042-2]
mal_ken	5	0.437[0.018-1.476]	0.211[0.009-0.712]
mal_gam	5	4.544[3.209-4.986]	2.19[1.547-2.404]
gam_ken	5	1.925[0.698-4.79]	0.928[0.337-2.309]
sa_mal	8	1.446[0.086-4.485]	0.872[0.052-2.705]
sa_ken	8	2.05[0.138-4.818]	1.236[0.083-2.905]
sa_gam	8	0.74[0.037-2.669]	0.446[0.022-1.609]
mal_ken	8	0.93[0.038-4.051]	0.561[0.023-2.443]
mal_gam	8	2.585[0.481-4.835]	1.559[0.29-2.916]
gam_ken	8	1.042[0.051-4.051]	0.628[0.031-2.443]
